# Mechanisms of obesity- and diabetes mellitus-related pancreatic carcinogenesis: a comprehensive and systematic review

**DOI:** 10.1038/s41392-023-01376-w

**Published:** 2023-03-24

**Authors:** Rexiati Ruze, Jianlu Song, Xinpeng Yin, Yuan Chen, Ruiyuan Xu, Chengcheng Wang, Yupei Zhao

**Affiliations:** 1 Department of General Surgery, Peking Union Medical College Hospital, Chinese Academy of Medical Sciences and Peking Union Medical College, 100730 Beijing, China; 2 Key Laboratory of Research in Pancreatic Tumors, Chinese Academy of Medical Sciences, 100023 Beijing, China; 3 Chinese Academy of Medical Sciences and Peking Union Medical College, No. 9 Dongdan Santiao, Beijing, China

**Keywords:** Cancer microenvironment, Gastrointestinal cancer, Endocrine system and metabolic diseases

## Abstract

Research on obesity- and diabetes mellitus (DM)-related carcinogenesis has expanded exponentially since these two diseases were recognized as important risk factors for cancers. The growing interest in this area is prominently actuated by the increasing obesity and DM prevalence, which is partially responsible for the slight but constant increase in pancreatic cancer (PC) occurrence. PC is a highly lethal malignancy characterized by its insidious symptoms, delayed diagnosis, and devastating prognosis. The intricate process of obesity and DM promoting pancreatic carcinogenesis involves their local impact on the pancreas and concurrent whole-body systemic changes that are suitable for cancer initiation. The main mechanisms involved in this process include the excessive accumulation of various nutrients and metabolites promoting carcinogenesis directly while also aggravating mutagenic and carcinogenic metabolic disorders by affecting multiple pathways. Detrimental alterations in gastrointestinal and sex hormone levels and microbiome dysfunction further compromise immunometabolic regulation and contribute to the establishment of an immunosuppressive tumor microenvironment (TME) for carcinogenesis, which can be exacerbated by several crucial pathophysiological processes and TME components, such as autophagy, endoplasmic reticulum stress, oxidative stress, epithelial-mesenchymal transition, and exosome secretion. This review provides a comprehensive and critical analysis of the immunometabolic mechanisms of obesity- and DM-related pancreatic carcinogenesis and dissects how metabolic disorders impair anticancer immunity and influence pathophysiological processes to favor cancer initiation.

## Introduction

As a multifactorial consequence of socioeconomic development, the prevalence of obesity and diabetes mellitus (DM) is booming in most parts of the world, regardless of the different landscapes among nations and regions.^1,2^ The detrimental outcomes and threats of obesity and DM include disability, a shortened life span, and many other critical conditions affecting both physical and mental health, whether acute, chronic, or even terminal.^1–6^ Some of the most vital sequelae of obesity and DM include various malignancies, including pancreatic cancer (PC).^7–9^ Despite its aggressiveness and lethality, the insidious symptoms of PC make employing applicable and sensitive screening methods difficult. However, etiological studies not only can help develop tools to detect PC at an early stage but also can provide decisive clues for effective prevention, which would undoubtedly benefit both cancer-free individuals and those with undiagnosed PC.

In this context, the accelerated rise in the prevalence of obesity and DM and the intimidating biological behaviors of PC have inspired tremendous explorations regarding their correlations. Beyond obesity and DM being causal factors of PC, PC, in turn, can also lead to an elevation of blood glucose and reduction of body weight, manifesting as the primary symptoms of occult malignancy.^10^ In terms of the carcinogenic effects of obesity and DM, many clinical studies and high-quality meta-analyses have confirmed the relationships of obesity and DM with PC, with basic studies focusing on the different aspects of these two most common metabolic disorders providing multitudinous insights to unveil the mechanisms behind the clinical evidence.

To date, the revealed mechanisms of obesity- and DM-related pancreatic carcinogenesis cover almost all of the immunometabolic alterations in these two diseases, which jointly reprogram systemic metabolism and remodel the local microenvironment of the pancreas, creating a perfect storm for the gradual initiation of PC. In brief, as both an endocrine and exocrine organ, the pancreas is not only the producer of hormones but also the receptor of many hormones. Thus, the changes in hormone levels and dysbiosis of the microbiome in obesity and DM inevitably lead to metabolic remodeling and pernicious accumulation of substantial nutrients and metabolites. These nutrients, metabolites, and other components within the reshaped tumor microenvironment (TME) provide precancerous and cancerous cells with mutagens, energy, hormones, and growth factors (GFs) and support the interactions between the surrounding cells and cancer cells via autocrine/paracrine signals and rewired metabolism while creating an inflammatory and immunosuppressive TME.^11^ As a result, the protective inflammatory/immune responses and various physiological processes collapse, and the carcinogenic microenvironment favors cancer initiation owing to continuously strengthened protumorigenic factors and compromised anticancer defense.

Although some previous studies have illustrated the correlations among obesity, DM, and PC, they were not entirely focused on the intricate mechanisms of the carcinogenic effects of obesity and DM. In addition, the exponential growth in the number of studies newly published on this topic also necessitates a comprehensive review of the key findings and questions. Beginning with a brief introduction of pancreatic carcinogenesis, we aim to provide a broad overview of the critical clinical and experimental discoveries through an in-depth look at the mechanisms of obesity- and DM-related pancreatic carcinogenesis, and we hope this overview will provide suggestions and guidance for experimental practice and research in this area.

## Pancreatic carcinogenesis

PC has a variety of histological classifications that differ in terms of PC development, biological behaviors, clinical features, and response to treatments, among which pancreatic ductal adenocarcinoma (PDAC) is predominant (>90%).^12^ Therefore, the carcinogenic process of PDAC will be briefly introduced as a representative.

In general, it takes more than 20 years to develop clinically detectable PC,^13^ giving an extensive time window for obesity and DM to promote carcinogenesis. The cellular origin of PDAC is still disputable, as both acinar and ductal cells are heterogeneous in their capacity to be transformed.^14,15^ Nevertheless, mutation in the proto-oncogene *KRAS* is the most common event and the primary regulator of the initiation of PC,^16,17^ abetted by sequential inhibition or inactivation of tumor suppressor genes during the progression of precancerous lesions [pancreatic intraepithelial neoplasia (PanIN)], including cyclin-dependent kinase inhibitor 2A *(CDKN2A)*, tumor suppressor p53 *(TP53)*, and SMAD family member 4 *(SMAD4)*.^9^ In other words, a single mutation in *KRAS* cannot cause cellular transformation without additional genetic alterations, which jointly sabotage cell identification, KRAS signaling, the cell cycle, apoptosis, senescence, DNA repair, and cellular metabolism.^15^ PanINs are divided into grades 1~3, and only PanIN3 (high-grade dysplasia) is the true precursor of cancer in situ.^18^ Within these neoplasms, the cellular heterogeneity includes metaplastic epithelia and low-grade dysplasia, with many tuft cells that mediate inflammatory responses in other glandular tissues.^19^ Neuroendocrine PanINs respond to neuronal signals to enhance lesion growth^20^ while frequently delaminating and entering the surrounding stroma,^21^ facilitating metastasis even in the absence of a carcinoma.

The desmoplastic stroma of PDAC harbors pancreatic stellate cells (PSCs), activated cancer-associated fibroblasts (CAFs), tumor-associated macrophages (TAMs), other immune cells, cancer cells, and the microvasculature.^15^ This immunosuppressive microenvironment is characterized by a disrupted inflammatory response and an aberrant extracellular matrix (ECM), shielding cancer cells from immune surveillance and attack.^22,23^ As a critical part of the TME, TAMs play an essential role in the inflammatory environment that promotes pancreatic carcinogenesis. First, oncogenic *KRAS* drives proinflammatory signaling in precancerous lesions by activating the nuclear factor-κB (NF-κB), signal transducer and activator of transcription 3 (STAT3), and glycogen synthase kinase 3 (GSK3)/nuclear factor of activated T cells (NFAT) pathways,^24–26^ where inflammatory macrophages promote acinar cell dedifferentiation, acinar-to-ductal metaplasia (ADM), and the formation of precancerous lesions by secreting provocative mediators.^27^ Next, inflammatory macrophages upregulate tissue inhibitors of metalloproteinases or matrix metalloproteinases (MMPs) and remodel the acinar microenvironment by promoting ADM.^27^ However, the local inflammation caused by mutant *KRAS* alone is insufficient for pancreatic carcinogenesis, which requires additional inflammatory fuels and genetic changes.^28^ What are the critical drivers of fibrogenesis in the microenvironment? The inflammatory cytokines secreted in PanIN1 lesions initiate the phenotypic switch of macrophages and are vital in suppressing inflammation and promoting the growth of the lesions.^29^ Finally, TAMs promote the epithelial-to-mesenchymal transition (EMT), invasiveness, and metastasis of PDAC through the immunosuppression of T cells and the regulation of fibrinogenesis, vascularization, and angiogenesis.^15^

**Fig. 1:**
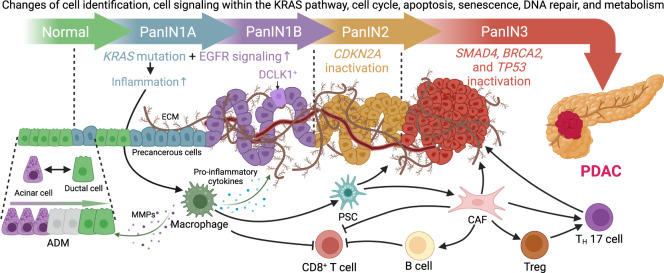
Progression and microenvironment of PanINs that favor the formation of PDAC. Ductal cells can transdifferentiate into acinar cells under normal conditions as a compensatory regenerative process to maintain the proper function of the pancreas. Meanwhile, the highly plastic acinar cells can also be turned into ductal cells through the metaplastic process called ADM when stimulated by inflammatory macrophages via the secretion of MMPs, and they maintain their ductal phenotype in the presence of oncogenic *KRAS* mutation, followed by enhanced EGFR signaling and sequential inactivation of tumor-suppressive genes, such as *CDKN2A*, *TP53, BRCA2*, and *SMAD4*, to form a carcinoma in situ. Initially, oncogenic *KRAS* magnifies proinflammatory signaling in macrophages and promotes ADM by producing MMPs while secreting inflammatory cytokines into the microenvironment. Some serine/threonine-protein kinase DCLK1^+^ cells of acinar origin are also formed during low-grade PanIN lesions, such as PanIN1A, PanIN1B, and PanIN2, putatively serving as progenitor cells with cancer stem cell functions. Meanwhile, macrophages activate PSCs and change their morphology into CAFs, which enhance the desmoplastic reaction and ECM production, increasing tissue tension and creating a hypoxic microenvironment within the PanINs that is made up of abundant precancerous metaplastic epithelia and tuft cells. Furthermore, CAFs can activate immunosuppressive B cells, Tregs, and T_H_17 cells and collaboratively sabotage the anticancer immunity of CD8^+^ T cells with macrophages. During this process, precancerous cells are transformed by strengthened KRAS signaling. The aberrance of the cell cycle, apoptosis, senescence, DNA repair, and metabolism in this immunosuppressive microenvironment jointly favors the formation of PDAC. ADM acinar-to-ductal metaplasia, CAF cancer-associated fibroblast, CDKN2A cyclin-dependent kinase inhibitor 2A, DCLK1 doublecortin-like kinase 1, ECM extracellular matrix, EGFR epidermal growth factor receptor, KRAS Kirsten rat sarcoma viral oncogene homolog, MMPs matrix metalloproteinases, PanIN pancreatic intraepithelial neoplasia, PDAC pancreatic ductal adenocarcinoma, PSC pancreatic stellate cell, SMAD4 SMAD family member 4, TP53 tumor suppressor p53, Tregs T helper cells, T_H_ helper T. This figure was adapted from a previous publication^15^

Other components of the TME also participate in the initiation of PDAC. CAFs from multiple origins are responsible for producing an ECM that contains various components, and PSCs are the main contributors to the desmoplastic reaction.^30^ In the normal pancreas, quiescent PSCs are activated during acute or chronic inflammation^31^ and change their morphology into myofibroblast-like cells to enhance ECM production.^32^ Consequently, the abundance of CAFs and a collagen- and hyaluronic-rich ECM promotes vasculature and increases tissue tension, creating a hypoxic microenvironment^33^ and altering tumor metabolism,^34^ which is essential for carcinogenesis.^15^ Furthermore, in contrast to the suppression of T-cell function in PDAC that leads to cancer cell proliferation, immune evasion, and metastasis, B cells enhance cell proliferation, inhibit antitumor immunity, and promote the progression and metastasis of cancer in multiple ways^15^ (Fig. 1).

In summary, the mutation of *KRAS* is one of the earliest events in pancreatic carcinogenesis, which simultaneously activates intrinsic pathways by inducing inflammation and promoting interactions among acinar cells, ductal cells, immune cells, and fibroblasts that jointly favor an immunosuppressive and fibroinflammatory microenvironment suitable for the promotion of the plasticity of neoplastic cells at all stages of tumor progression.^27^ Regardless of the differences in pathological characteristics, microenvironmental aberrance of the inflammatory response, immune abnormalities, and fibrosis are commonly present in obesity, DM, and PC. Thus, it can be assumed that these similarities could be the main drivers of pancreatic carcinogenesis. For further reference, the carcinogenic process of PDAC is reviewed in more detail here.^15^

## Mechanisms of obesity- and DM-related pancreatic carcinogenesis

Cancer cells are persistently influenced by the TME, which is predominantly shaped by the metabolic abnormalities of the host, providing beneficial hormones, GFs, nutrients, and metabolites and supporting the interactions between the surrounding cells and cancer cells via autocrine and paracrine signals while creating an inflammatory and immunosuppressive TME in the context of obesity and DM. The systemic immunometabolic abnormalities caused by obesity and DM are extremely complicated. Reprogrammed metabolism affected by internal or external factors and rewired glucose, amino acid, and lipid metabolism and metabolic crosstalk within the TME is critical in pancreatic carcinogenesis. As an endocrine and exocrine organ, the pancreas is not only the producer of hormones but also the receptor of many hormones. The changes in hormone levels and dysbiosis of the microbiome in obesity and DM inevitably lead to metabolic remodeling and pernicious accumulation of nutritional metabolites. At the same time, the reshaped metabolism reprograms inflammatory/immune responses and various physiological processes that are supposed to be anticarcinogenic. In this context, the aberrant microenvironment breeds pancreatic carcinogenesis owing to continuously strengthened cancer-promoting factors and the collapse of anticancer defense.

### Nutrients and metabolites

#### High-fat diet and lipids

The contributive impact of a high-fat diet (HFD) on pancreatic carcinogenesis has been known for over 20 years,^35^ and not only can an HFD contribute to pancreatic carcinogenesis through the induction of obesity or DM, but it also has been shown to affect the carcinogenetic processes directly in different animal models. It was shown in *P48*^*+/Cre*^*; LSL-KRAS*^*G12D*^ *(KC)* mice that an HFD significantly increased the incidence and progression of precancerous lesions of PC via sustained inflammation and dysregulated autophagy.^36^ An HFD can also contribute to pancreatic carcinogenesis by augmenting pancreatic fatty infiltration through its obesogenic effect in Syrian golden hamsters treated with N-nitrosobis (2-oxopropyl) amine (BOP).^37^ In addition, it was demonstrated in C57BL/6 mice fed an HFD that this dietary pattern can be carcinogenic by stimulating inflammation via gut microbiome (GM) alteration, which occurs before the potential influence of circulating inflammatory cytokines.^38^ The effects of an HFD on inflammation and GM composition can also enhance the progression of carcinogen-induced PC in C57BL/6 mice.^39^ In three studies using different genetically engineered mouse models (GEMMs), an HFD was shown to increase inflammation, fibrosis, and PanIN lesions while promoting the transformation of precancerous lesions into more aggressive PDAC through enhanced cyclooxygenase 2 (COX2)-activated KRAS signaling,^40^ aerobic glycolysis,^41^ RAS activity, and reduced expression of fibroblast growth factor 21 (FGF21).^42^ In addition to promoting pancreatic carcinogenesis via the dysregulation of autophagy, increased genetic alterations in PanINs^36^ and reduced DNA repair in precancerous cells,^43^ an HFD also exacerbates tumor growth, angiogenesis, and EMT while decreasing apoptosis.^44^ In particular, the tumor-promoting effect of an HFD was suggested to be regulated by endogenous cholecystokinin (CCK),^45,46^ and this effect could not be ameliorated by physical exercise.^47^ Other physiological impacts of an HFD on PC include enhanced lipid metabolism, altered oxidative stress, extensive central necrosis, and lipid accumulation.^48^ In addition, diet and obesity, in a setting of an HFD, were demonstrated to promote pancreatic carcinogenesis via phosphatidylinositol 3-kinase (PI3K)/protein kinase B (AKT) signaling.^49^

Some lipids are also involved in pancreatic carcinogenesis related to obesity and DM. Obesity and DM are common risk factors for dyslipidemia characterized by elevated circulating levels of very-low-density lipoprotein (VLDL), low-density lipoprotein (LDL), and triglycerides (TAGs). In contrast, the level of high-density lipoprotein (HDL) is decreased.^50^ Dyslipidemia has long been recognized as a risk factor for PC,^51,52^ and high dietary cholesterol, which contributes to dyslipidemia, also increases the risk of PC.^53^ In addition, previous research has demonstrated that dyslipidemia contributes to pancreatic carcinogenesis by deteriorating pancreatic fatty infiltration.^54^ However, while conflicting results exist regarding the effects of pharmacological treatment of dyslipidemia (mainly statins) on the risk of PC,^55–57^ animal studies have suggested a detrimental role of statins in the development of PC.^58^ In contrast, atorvastatin, another 3-hydroxy-3-methyl glutaryl coenzyme A (HMG-CoA) reductase inhibitor, was demonstrated to suppress pancreatic carcinogenesis and prolong the survival of rodents with PC.^59^

Cholesterol is an essential molecule that maintains the normal function of cellular membranes, and it is a precursor for synthesizing steroid hormones, oxysterols, and bile acids (BAs), acting as a signaling molecule regulating the cell cycle and the modification and synthesis of proteins.^60^ In contrast to the speculation that cholesterol induces a higher PC risk, total serum cholesterol was inversely related to the risk of PC independent of statin use.^61^ Notably, LDL promotes the proliferation of PC cells by activating the STAT3 pathway while upregulating the expression of multiple oncogenic genes.^62^ This can be partly explained by the role of interleukin 6 (IL-6)/Janus kinase (JAK)/STAT3 signaling in cancer,^63^ as IL-6 is a proinflammatory and protumorigenic cytokine capable of reducing the level of total serum cholesterol.^64^ PC cells are highly dependent on profoundly activated cholesterol uptake, which results in an increased influx of cholesterol and overexpression of the LDL receptor (LDLR),^51^ a major site that transports LDL, VLDL, and VLDL into the cells,^65^ increasing cell proliferation and activating the extracellular signal-regulated kinase (ERK) 1/2 pathway.^66^ In *TP53*-mutant PDAC cells, sterol O-acyltransferase 1 (SOAT1) sustains the mevalonate pathway by converting cholesterol to inert cholesterol esters, thereby preventing the negative feedback elicited by unesterified cholesterol, which promotes cell proliferation in vitro and tumor progression in vivo.^67^ Moreover, acyl-CoA cholesterol acyltransferase-1 (ACAT-1) was found to enhance the esterification and accumulation of cholesterol in human PC specimens and cell lines, suppressing apoptosis and supporting tumor growth.^68^

As mentioned above, cholesterol is a precursor for progesterone, estrogen, and androgen synthesis, which implies that cholesterol may contribute to pancreatic carcinogenesis by influencing the levels of sex hormones (which will be addressed later). In addition to its direct effects on the synthesis of steroid hormones, cholesterol is also metabolized into biologically active oxysterols. Oxysterols also have multiple functions, such as affecting membrane fluidity, regulating the sterol regulatory element-binding protein (SREBP) signaling pathway, and activating several nuclear receptors, such as retinoic acid receptor-related orphan receptors (RORs), farnesoid X receptor (FXR), pregnane X receptor (PXR), estrogen receptors (ESRs), and liver X receptors (LXRs).^69,70^ Among them, cholesterol metabolism is under the strict regulation of SREBPs^71,72^ and LXRs,^73^ which decrease cholesterol uptake via LDLR and increase cholesterol efflux.^74^ SREBPs are transcription factors that activate the transcription of genes enhancing cholesterol synthesis and uptake. Despite the primary regulator of cholesterol homeostasis being SREBP-2,^75^ the SREBP-1 pathway is essential for the growth, viability, and proliferation of PC cells.^76,77^ LXRs, members of a nuclear receptor family that regulate insulin secretion, cholesterol homeostasis, lipid metabolism, and inflammation, were shown to be dramatically elevated in PDAC.^78^ In contrast, LXR agonists can disrupt the proliferation, cell cycle progression, and colony formation of PDAC cells.^79,80^ Similarly, inhibiting the transcriptional activity of LXR with synthetic ligands reduces the proliferation of PDAC cells and tumor formation.^81^ Furthermore, as defects in DNA repair, increased DNA strand breaks, genomic instability, and gene mutagenesis are known to induce carcinogenesis, defective LXR/SREBP-1/polynucleotide kinase/phosphatase (PNKP) signaling was demonstrated to cause a reduction in both DNA repair and apoptosis in vivo and in vitro.^82^

In addition to mediating SREBPs and LXRs, other mechanisms are also involved in cholesterol-related pancreatic carcinogenesis. First, oxysterols have also been shown to increase inflammatory cytokines in macrophages,^83^ and tumor necrosis factor α (TNF-α) can affect the lipogenesis and inflammatory status of PDAC cells by regulating SREBP-1 and acetyl-CoA carboxylase (ACC),^84^ suggesting that cholesterol may also affect carcinogenesis via the inflammatory response. In addition, oxy186, a semisynthetic oxysterol analog as an inhibitor of Hedgehog (Hh) signaling acting downstream of Smoothened (Smo), was illustrated to suppress Hh signaling and the proliferation of PANC-1 cells.^85^ Finally, oxysterol binding protein-related protein 5 (ORP5) induces the expression of SREBP-2 to enhance the cholesterol synthesis pathway and activates histone deacetylase 5 (HDAC5) to promote the growth of PC cells.^86^

#### Fatty acids

Some fatty acids (FAs) are essential for mammals, and different FAs have distinct impacts on tumor growth. For example, omega-3 FAs and omega-6 FAs can be oxidized to acetyl-CoA, while omega-3 FAs have an anti-inflammatory effect both in vivo and in vitro, and omega-6 FAs have proinflammatory and protumorigenic properties in obesity.^87^ Consistent with epidemiological data suggesting an anticancer effect of diets high in omega-3 FAs, a preclinical study showed that an omega-3-FA-enriched diet suppressed pancreatic carcinogenesis via reduced phosphorylated AKT (pAKT), whereas an omega-6-FA-enriched diet augmented tumor formation.^88^ In patients with obesity, high levels of free fatty acids (FFAs) can activate preadipocytes and inflammatory cells by inducing Toll-like receptor 4 (TLR4) signaling.^89^ It was also shown that lipid metabolism and fatty acid oxidation (FAO) are implicated in pancreatic carcinogenesis initiated from intraductal papillary mucinous neoplasms (IPMNs).^90^

#### Glucose

Given the long period of pancreatic carcinogenesis, patients with obesity and DM are often asymptomatic for decades. Nevertheless, many of these patients suffer from the gradual development of glucose intolerance and hyperglycemia before cancer diagnosis. Many epidemiological studies have concluded that type 1 DM (T1DM) and type 2 DM (T2DM) increase the risk of PC in both sexes.^8,91^ Epidemiological data have also showed that hyperglycemia in the first few years, commonly known as new-onset DM, induces a higher PC risk than long-standing DM,^92^ whereas studies on *LSL-Kras*^*G12D/+*^*; LSL-Trp53*^*R172H/+*^*; Pdx-1-Cre* (*KPC*) mice did not show a relationship of paraneoplastic DM and pancreatic carcinogenesis.^93^ Hyperglycemia, as a hallmark of DM, provides cancer cells with excessive energy to stimulate their proliferation and accelerate the progression of carcinogenesis. Interestingly, cancer cells tend to use glycolysis instead of efficient ATP production for their expansion, the so-called Warburg effect,^94^ enabling cancer cells to survive in nutrient-deficient conditions.^95^ Metabolically, mechanisms connecting hyperglycemia and cancer include lipotoxicity and glucose-associated pathways such as autoxidation, oxidative phosphorylation, glycosylation, the glycosamine pathway, and the Hippo-Yes-associated protein (YAP) pathways,^96^ with the dysfunction of these pathways increasing reactive oxygen species (ROS) and weakening DNA stability in β-cells.^97^

Beyond directly accelerating PC development by providing excessive glucose to cancer cells, hyperglycemia also promotes cell proliferation via the induction of epidermal growth factors (EGFs) and their receptors (EGFRs) while causing endothelial dysfunction and promoting angiogenesis.^98^ In addition, multiple signaling pathways can be aberrantly activated in hyperglycemia. Activated NF-кB and p38 MAPK signaling in response to cellular stress and chronic inflammation under hyperglycemic conditions augments the proliferation and apoptosis of PC cells by enhancing the secretion of proinflammatory cytokines and the paracrine effects of vascular endothelial growth factor (VEGF), promoting EMT, cell growth and PC development.^98^ In addition to ECM remodeling and angiogenesis,^97^ hyperglycemia also promotes EMT^99,100^ and the stemness of precancerous cells to promote pancreatic carcinogenesis through the activation of transforming growth factor β (TGF-β) signaling.^101^ Moreover, under hyperglycemic conditions, cellular O-GlcNAcylation can be significantly elevated in pancreatic cells that exhibit lower phosphofructokinase (PFK) activity, which compromises ribonucleotide reductase (RNR) activity and leads to deficiency in dNTP pools, enhancing genomic DNA alterations with concurrent *KRAS* mutations and cellular transformation. All these changes induce the initial oncogenic *KRAS* mutations in pancreatic cells to trigger carcinogenesis.^102^

#### Advanced glycation end products and their receptors

**Fig. 2:**
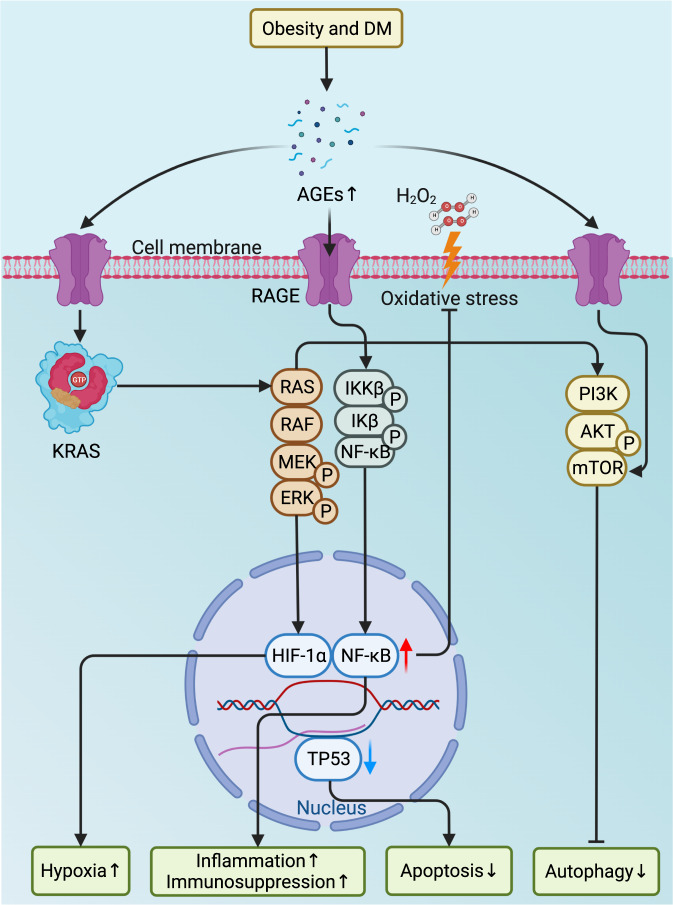
The roles of AGEs and RAGEs in pancreatic carcinogenesis. The production of AGEs is drastically increased in obesity and DM, and the binding of AGEs to RAGEs activates MAPK and NF-κB signaling and increases the transcription of HIF-1α and NF-κB, which prevents cell death from oxidative stress and creates a hypoxic microenvironment while promoting proinflammatory signaling to exacerbate inflammatory reactions and recruit immunosuppressive MDSCs to diminish anticancer immunity. In addition to the decreased apoptosis due to the decline in the transcription of *TP53* following the activation of KRAS signaling, the enhanced PI3K-AKT signaling and the direct activation of mTOR by RAGEs mitigate autophagy to improve the proliferation and survival of cancer cells, thereby promoting pancreatic carcinogenesis. AGEs advanced glycation end products, AKT protein kinase B, DM diabetes mellitus, ERK extracellular signal-regulated kinase, HIF-1α hypoxia-inducible factor 1 subunit α, IKKβ inhibitor of nuclear factor-κB (NF-κB) kinase subunit β, Ikβ inhibitor of NF-κB subunit β, KRAS Kirsten rat sarcoma viral oncogene homolog, MAPK mitogen-activated protein kinase, MDSCs myeloid-derived suppressor cells, MEK mitogen extracellular kinase, mTOR mammalian target of rapamycin, NF-κB nuclear factor-κB, P phosphorylation, PI3K phosphatidylinositol-3-kinase, RAF Raf proto-oncogene, RAGE receptor of AGEs, TP53 tumor suppressor p53

Referring to a heterogeneous class of molecules resulting from a nonenzymatic reaction of the oxo group of carbohydrates and the free amino group of amino acids, lipids, nucleic acids, or their combinations, advanced glycation end products (AGEs) are excessively produced and accumulate in hyperlipidemic and hyperglycemic conditions such as obesity, DM, and their comorbidities.^103,104^ Receptors of AGEs (RAGEs) belong to the immunoglobulin superfamily and are multiligand transmembrane receptors present on various cells.^105,106^ To date, ample evidence has demonstrated the potential contribution of AGE/RAGE crosstalk to pancreatic carcinogenesis through different mechanisms (Fig. 2). First, RAGEs prevent cell death and apoptosis by suppressing *TP53* transcription and autophagy to improve the proliferation and survival of PC cells.^107,108^ Second, RAGEs promote the recruitment and retention of myeloid-derived suppressor cells (MDSCs) in the TME to protect pancreatic neoplasms from the antitumor immune response.^109,110^ In addition, since NF-κB is essential for inflammatory signaling in PanINs,^111^ the binding of NF-κB to RAGEs maintains the longstanding inflammatory state preferable for carcinogenesis.^112^ Meanwhile, as hypoxia induces NF-κB-dependent and hypoxia-inducible factor 1 subunit α (HIF-1α)-independent RAGE expression in PC cells, along with enhanced interaction between RAGEs and mutant *KRAS* facilitating the transcriptional activity of HIF-1α, the activation of NF-κB signaling deteriorates hypoxia by enhancing HIF-1α activation.^113^ Finally, in an NF-κB-dependent manner, RAGEs prevent PC cells from H_2_O_2_-induced oxidative injury during oxidative stress.^114^ Overall, RAGEs support carcinogenesis by creating an immunosuppressive TME while promoting the survival of PC cells. Interestingly, while dietary consumption of AGEs was suggested to modestly increase the risk of PC in men,^115^ others failed to confirm the association between AGEs/RAGEs and PC risk.^116^

#### Bile and bile acids

Being influenced by lifestyle factors such as smoking, alcohol consumption, and dietary habits, bile and BAs are also closely related to the pathogenesis of obesity and DM.^117,118^ While heavy alcohol consumption alters the levels of BAs in the blood and intestines, which affects the GM, influences intestinal permeability, and induces systemic inflammation, intracellular signaling pathways are also activated in pancreatic epithelial cells owing to low-dose exposure to BAs.^117^ As a mutagen associated with PC, cigarette smoke stimulates the activation of mutated *KRAS* as well as that of other mutated proteins, such as those encoded by *TP53*, *COX2*, *SMAD4*, and *p16INK4A*.^117^ Mechanistically, nicotine promotes pancreatic carcinogenesis by increasing the secretion of gastric acid while disrupting the secretion of BAs.^117^ Concerning the effects of dietary habits, the physiological function of BAs is to promote the absorption of dietary fat and fat-soluble vitamins as a mediator of cholesterol metabolism. Thus, the levels of BAs are drastically elevated in individuals with an HFD, as dietary fat significantly stimulates the secretion of BAs. Although the pancreas does not make direct contact with BAs, the fact that nearly 60% of PC tumors occur in the head of the pancreas adjacent to the bile tracts implies a probability that BAs may play a role in pancreatic carcinogenesis, as previous studies have confirmed the association between BAs and cancers of multiple sites.^117^

FXR is a critical mediator of BA synthesis and metabolic control, and various preclinical studies have concluded that FXR is involved in the initiation of multiple cancers.^119–121^ FXR was significantly increased in PC cell lines and was found to be the regulator of the focal adhesion kinase (FAK)/JUN N-terminal kinase (JNK)/Mucin (MUC) 4 signaling pathway.^122^ Likewise, in both PDAC cell lines and human samples, it was found that bile accelerates carcinogenesis through the overexpression of MUC4.^123^ In addition to their contribution to pancreatic carcinogenesis via insulin resistance [or elevated insulin-like growth factor 1 (IGF-1) signaling], hyperinsulinemia, and the disruption of the GM in obesity and DM, BAs also elevate the risk of PC via gallstones, pancreatobiliary maljunction, and chronic pancreatitis.^117,124^ Moreover, BAs can have much more direct and local effects on carcinogenesis. For example, BAs induce cell membrane perturbations by disrupting the redistribution of membrane cholesterol and promoting cell proliferation with their mitogenic impact while reducing apoptosis.^117^ They also enhance inflammatory reactions and activate signaling pathways closely related to pancreatic carcinogenesis, such as Erb-B2 EGFR, mitogen-activated protein kinase (MAPK), and STAT3 signaling.^117^

#### Amino acids

Apart from being involved in the pathogenesis of obesity and DM,^125^ amino acids are vital for the survival of all cells and rewired metabolism in cancers, and they play distinct roles within the carcinogenic TME, serving as energy sources, regulators of epigenetics and immune responses, and therapeutic targets.^126^ Preclinical research has demonstrated that macropinocytosis, a highly conserved endocytic process transporting extracellular fluid and its contents into oncogenic Ras-transformed cells, supports the growth of these cells through the internalization of amino acids, including glutamine, that are translated into proteins.^127^ Emerging evidence has suggested that different amino acids participate in pancreatic carcinogenesis. It was shown in patients undergoing pancreatic resection that the circulating levels of branched-chain amino acids (BCAAs) were correlated with the dysplastic grades of IPNM, a high-risk precancerous lesion.^128^ A recent study indicated that BCAA uptake promotes PDAC development, while BCAA catabolism is impeded in PDAC tissue, indicating that BCAA uptake could be a promising therapeutic target for the treatment of PDAC.^129^ Isoleucine, one of the BCAAs, was associated with an increased risk of PC in women with long-term obesity.^130^ It was demonstrated in *KC* mice that KRAS stabilizes BCAA transaminase 2 (BCAT2) via the regulation of spleen tyrosine kinase (SYK) and E3 ligase tripartite-motif-containing protein 21 (TRIM21) to enhance BCAA uptake and mitochondrial respiration, which fosters the progression of PanIN.^131^ Similarly, TRIM2 was shown to promote PC progression by activating ROS-related nuclear factor (erythroid-derived 2)-like 2 (NRF2)/antioxidant response element (ARE) signaling and the integrin/FAK pathway.^132^

The synthesis of amino acids and proteins can also fuel pancreatic carcinogenesis. The enhanced mTOR-dependent serine synthesis and upregulation of DNA methylation due to the loss of liver kinase B1 (LKB1, also known as STK11) synergize with KRAS activation to promote pancreatic carcinogenesis in GEMMs and primary pancreatic epithelial cells.^133^ Similarly, protein synthesis is also involved in pancreatic carcinogenesis. In Ras-driven cancers such as PC, the guanosine triphosphatase (GTPase) activity of eukaryotic elongation factor 1A (eEF1A) catalytically increased by methyltransferase-like 13 (METTL13) augments protein production in vitro, and METTL13 dimethylation of eEF1A lysine 55 (eEF1AK55me2) enhances translation and protein synthesis to promote carcinogenesis in vivo.^134^

Additionally, amino acid modification can also contribute to pancreatic carcinogenesis. For example, the deregulation of lysine methylation signaling has been shown to be a common pathogenic factor in cancers, making inhibitors of several histone lysine methyltransferases (KMTs) ideal chemotherapeutics.^135^ Among these KMTs, SET and MYND domain-containing protein 3 (SMYD3) was suggested to promote carcinogenesis in mouse models of PDAC via the methylation of MAP kinase MAP3K2 at lysine 260 and subsequently activate RAS signaling.^135^

#### Acetyl-coenzyme A

Acetyl-coenzyme A (acetyl-CoA) is a central metabolic intermediate of the tricarboxylic acid (TCA) cycle and the primary regulator of cellular metabolism. Acetyl-CoA affects the activity and specificity of enzymes and the acetylation profile of proteins, thereby controlling vital cellular processes such as energy balance, mitosis, and autophagy that are implicated in the development of obesity and DM.^136^ Recent studies have also illustrated the roles of acetyl-CoA in pancreatic carcinogenesis. It was shown that the elevated levels of acetyl-CoA induced by adenosine triphosphate (ATP)-citrate lyase (ACLY) in *KRAS*-mutant acinar cells promoted ADM and tumor formation via histone acetylation and the mevalonate pathway.^137^ Fueled by the phosphorylation of acyl-CoA thioesterase (ACOT) at S392 by AKT, the accumulation of ACOT catalyzes the hydrolysis of acyl-CoA thioesters and produces nonesterified FAs and coenzyme A (CoA), which provides excessive CoA to promote the proliferation and tumor formation of PDAC cells.^138^

As mentioned above, many dysregulated nutrients and metabolites in obesity and DM can promote pancreatic carcinogenesis. However, most of these findings were based on observations in different animal models, and the scarcity of clinical evidence warrants more future studies to validate these impacts in humans.

### Endocrine and exocrine factors

Long known as being vital for the normal functioning of the pancreas, the exocrine-endocrine axis is responsible for the extensive regulation of physiological and pathophysiological processes. The pancreas is a hormone-producing organ and a target of many hormones itself. Various gastrointestinal (GI) hormones/peptides and sex hormones have been suggested to be involved in pancreatic carcinogenesis.

#### Gastrin and CCK

Gastrin, a peptide released by G cells in the pyloric antrum of the stomach, duodenum, and pancreas, stimulates the secretion of gastric acid (HCl) by the parietal cells of the stomach and aids in gastric motility; gastrin also plays a critical role in the development of the GI tract and the regulation of satiety. Usually, gastrin is not expressed in the adult pancreas but surprisingly reappears in PanINs.^139^ Patients with pernicious anemia and elevated serum gastrin levels have an increased incidence of pancreatic neoplasia.^140^ It was also shown that gastrin promotes the growth of several human PC cell lines in an autocrine manner^141,142^ as a ligand binding to CCK B receptor (CCK-RB) to participate in pancreatic carcinogenesis.

Secreted by a unique species of enteroendocrine cells (EECs) called I cells, CCK responds to meal digestion, regulates satiety, and controls blood glucose by affecting hepatic glucose production and gastric emptying,^143^ and the dysregulation of CCK signaling can contribute to the pathogenesis of obesity and T2DM.^143^ In addition, despite neither gastrin nor CCK being mutagenic, they can accelerate the progression of existing *KRAS* mutations and PanIN lesions.^144^ Gastrin and CCK were found to significantly enhance the proliferation of PC cells in vitro,^142,145^ and the high level of CCK in the blood induced by dietary fat was suggested to promote the growth of an established PC tumor in animal models.^146^

The carcinogenic effect of gastrin and CCK lies in the autocrine mechanism of gastrin sustaining tumor growth through enhanced transcription in cancer cells by activating CCK-RB,^147^ and the expression of gastrin is ubiquitous and essential for carcinogenesis and cancer progression in PC.^148^ In contrast, CCK is not thought to be expressed in the pancreas.^149^ However, it has been shown that the aberrant expression of *Cck* in pancreatic β-cells in response to obesity enhances the proliferation and ductal transformation of acinar cells to promote *Kras*-driven pancreatic carcinogenesis, indicating that obesity-associated changes in the TME implicate endocrine-exocrine signaling in PDAC development.^150^ Nevertheless, the expression of gastrin and CCK is detectable in PC tissues,^151^ although CCK produced by the tumor is likely to be inefficient in influencing the growth of PC.^152^ Therefore, it can be hypothesized that the carcinogenic effect of gastrin and CCK along with their presence in PC result from the re-expression of endogenous gastrin through an autocrine mechanism.^153^

There are two classic types of CCK receptors, named CCK-RA and CCK-RB,^154^ that are predominant in the normal pancreas of mice and humans, respectively.^155^ Regardless of its low abundance, the increase in CCK-RB is significantly related to the development of PC.^156^ In addition, a mutant of CCK-RB called CCK-RC (CCK-cancer receptor) is related to higher aggressiveness and shortened survival.^157^ For the intracellular signaling of CCK-RB in PC, the activation of CCK-RB or the splice variant CCK-RC triggers a conformational change in receptors and leads to the activation of various secondary messenger molecules responsible for the regulation of cell growth, proliferation, differentiation, migration and invasion, angiogenesis, and cell survival.^153^ In more detail, gastrin stimulation activates AKT phosphorylation, MAPK (including the four subgroups ERK1/2, JNKs, ERK5, and p38-MAPK) pathways, and cyclins through CCK-RB.^153^

As introduced, the inflammatory TME of obesity and DM is important in carcinogenic progression. It has been demonstrated that CCK receptors and CCK are essential in accelerating PanIN progression under inflammatory conditions.^144,155^ Furthermore, CCK receptors were found on PSCs,^158^ the nonepithelial component of the TME, with the activation of these receptors being suggested to promote desmoplasia in PC.^159^ Given the nonnegligible roles of gastrin and CCK in carcinogenesis, massive efforts have been made to target CCK/gastrin signaling pathways, and selective CCK-RB antagonist blockade and downregulation as well as the neutralization of the potent trophic effects of gastrin through nanotechnology and immunotherapy have been shown to be promising in several types of malignancies.^153^

#### Insulin resistance/hyperinsulinemia and IGF-1 axis disruption

Since insulin resistance is positively correlated with obesity and DM, elevated fasting serum insulin levels and insulin resistance are also associated with a higher risk of PC through a combined effect of IGFs.^160–162^ It was found that rather than hyperglycemia or pancreatic β-cell dysfunction, circulating markers of peripheral insulin resistance were independently associated with PC risk.^163^ In addition, nonfasting C-peptide levels were also shown to be associated with this risk.^164^ Synthesized by almost every organism tissue, IGFs consist of the insulin receptor (IR), IGF-1 receptor (IGF-1R), and IGF-2R, along with the ligands of insulin, IGF-1, and IGF-2 and the IGF-binding proteins (IGFBPs) that bind to IGF-1 and IGF-2, jointly regulating the growth, development, and survival of cells. IRs and IGFRs all belong to the receptor tyrosine kinase (RTK) family, which includes two different IRs and IGFRs, IR-A/IR-B and IGF-1R/IGF-2R, respectively. While IGF-1R is expressed in nearly all tissues, with the majority found to be IGF-1R/IR hybrids,^165^ IGF-2R is ubiquitously expressed and does not induce activation of the insulin-IGF signaling axis.^166^ Furthermore, the IGF signaling pathway consists of six IGFBPs and ten IGFBP-related proteins (IGFBP-RPs),^167^ and the complexity of this signaling pathway endows the insulin-IGF signaling axis with numerous modes of activation and intricate roles in pancreatic carcinogenesis.

Proinsulin can contribute to pancreatic carcinogenesis by inducing cell proliferation and migration through the ERK/p70S6K pathway.^168^ Insulin/IGF signaling regulates the development and function of the endocrine pancreas by controlling the function of β-cells, stimulating cell proliferation, and increasing cell mass and basal insulin production.^162^ Due to the dysregulation of IGFs in obesity and DM,^169^ as well as the overexpression of IGFs or IGF-1R in cancer cells, stromal cells exert neoplastic actions by promoting cell cycle progression and inhibiting apoptosis either directly or indirectly through preacquired oncogenic drivers.^162^ Mutations in *KRAS* and elevated insulin and IGF-1 levels can activate PSCs and thereby increase stromal fibrosis within the islets^162^ and peri-islet tissue.^170^ On the one hand, elevated insulin can increase IGFs by reducing IGFBPs.^171^ On the other hand, insulin and IGFs induce a variety of carcinogenetic effects on target cells and influence cell proliferation, apoptosis, angiogenesis, and lymphangiogenesis,^172^ which also implicates the PI3K/AKT/mTOR pathway in regulating cell growth and differentiation and the MAPK pathway in enhancing cell proliferation in obesity.^173^ Overall, IGF-1- and IGF-1R-mediated signaling promote cell proliferation and expression of angiogenic factors and decrease apoptosis in obesity-related pancreatic carcinogenesis.^174^

Other mechanisms of insulin contributing to DM-related PC include the upregulation of the expression of transgelin-2, which binds with SREBP-1 to alter lipid metabolism.^175^ In addition, insulin regulates glucose uptake in target tissues while acting as a mitogen on PC cells. Beyond its mitogenic effects, IGF-1 promotes PC growth by enhancing angiogenesis and EMT while inhibiting apoptosis.^98^

#### Sex hormones

The latest cancer statistics suggest that men suffer from a higher incidence of PC than women,^176^ indicating that there could be an impact of sex hormones on pancreatic carcinogenesis. However, some have proposed that this higher incidence is a consequence of the many environmental factors that men are more likely to be exposed to, such as smoking and alcohol. So, do sex hormones have nothing to do with the discrepancies in the incidence of PC between the sexes?

**Fig. 3:**
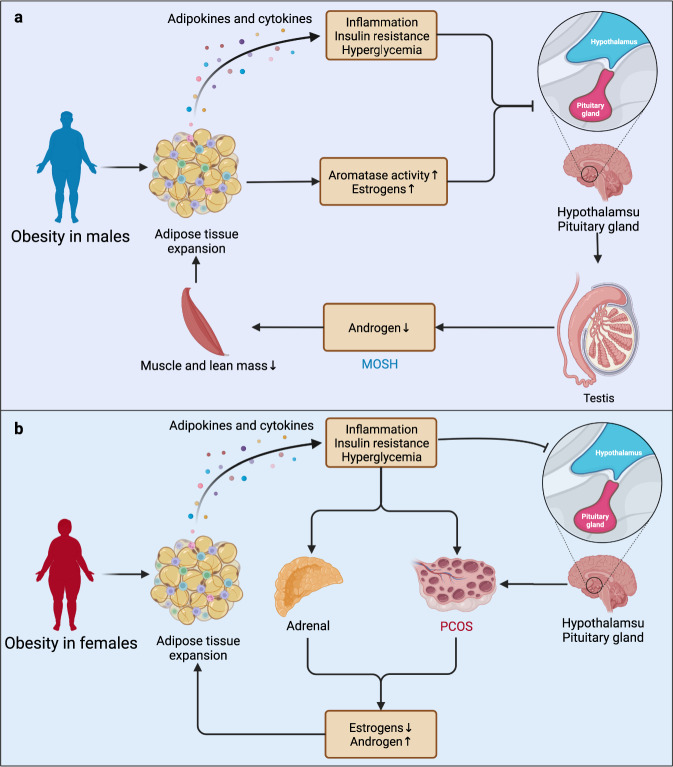
Cyclical relationships between obesity and dysregulated sex hormone levels in both sexes. The excessive accumulation and expansion of adipose tissue disrupts the secretion of metabolic and inflammatory adipokines and cytokines, eventually causing systemic inflammation, insulin resistance, and hyperglycemia. **a** In men, metabolic disorders along with increased aromatase activity and estrogen levels induce an inhibitory effect on the secretion of gonadotropin from the hypothalamus and pituitary gland, suppressing the production of androgen from the testes and resulting in MOSH and the exacerbation of obesity. **b** In contrast, the inhibitory effect of the hypothalamus and pituitary gland on the ovaries and the influence of systemic inflammation and metabolic disorders on the adrenals and ovaries lead to the elevation of androgen, resulting in PCOS and hyperandrogenism in women. MOSH male obesity-associated secondary hypogonadism, PCOS polycystic ovary syndrome

Before answering that question, we should keep in mind that a vicious cyclical relationship exists between obesity and sex hormones (Fig. 3). Obesity can cause hypogonadism, which in turn can result in or exacerbate obesity and other metabolic disorders, such as insulin resistance and DM.^177–179^ In short, the impact of obesity on gonadal function involves insulin resistance at the hypothalamic and pituitary levels. The inhibitory effect on gonadotropin secretion of inflammatory mediators secreted by adipose tissue leads to sexual dimorphism with androgen deficiency, causing male obesity-associated secondary hypogonadism (MOSH). In contrast, excessive androgen leads to polycystic ovary syndrome (PCOS) and idiopathic hyperandrogenism in women, jointly contributing to metabolic disorders and the dysfunction of other organs.^179^ Recently, the correlation between PCOS and the risk of PC has been confirmed by a case‒control study.^180^ Conversely, intentional weight loss and other weight-lowering interventions effectively ameliorated obesity-related hypogonadism.^179^ Consistently, a recent study suggested that hormone therapy is also an ideal way to prevent or reverse T2DM in patients with obesity.^181^

**Fig. 4:**
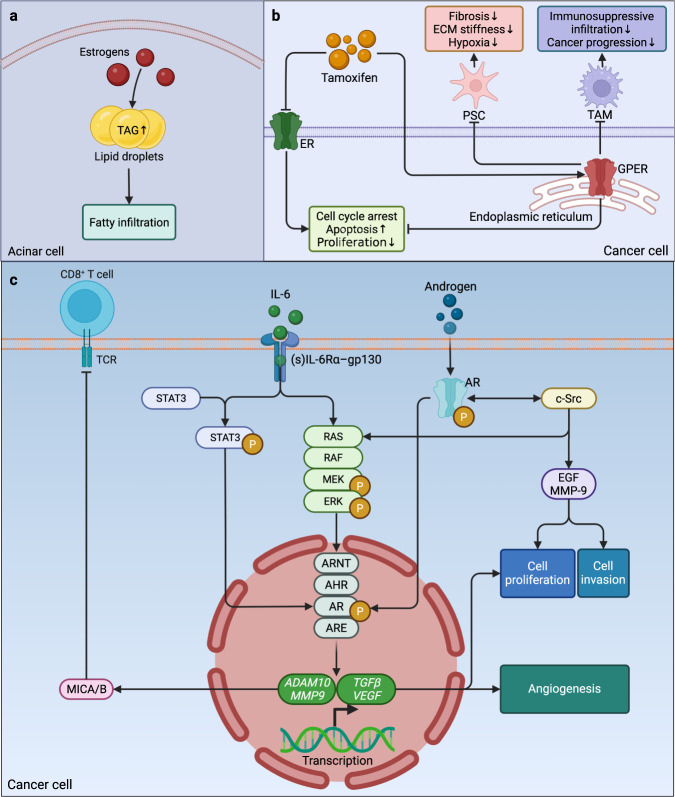
Sex hormones and pancreatic carcinogenesis and cancer progression. **a** In the cytoplasm of pancreatic acinar cells, the increased estrogen levels lead to the elevation of TAGs and total lipids in the pancreas, contributing to fatty infiltration. **b** Tamoxifen can play an anticancer role by antagonizing estrogen receptors and agonizing GPER, and the latter can mitigate fibrosis and hypoxia in the TME by targeting PSCs, while it also ameliorates the immunosuppressive infiltration of macrophages and hinders cancer progression. **c** According to the description of Kanda et al. ^195^, the tumorigenic cytokine IL-6 can activate both STAT3 and MAPK signaling in PC cells, while extracellular androgen and oncogenic c-Src can also enhance AR and MAPK signaling and trigger the transactivation of nuclear ARs. Meanwhile, AHR, ARNT, and ARE interact with AR in a testosterone-dependent manner and translocate into the nucleus to increase the transcription of *ADAM10*, *MMP9*, *TGFβ*, and *VEGF*. ADAM10 and MMP-9 increase the expression of MICA and MICB and hamper the immune response of NK cells and T cells against cancer cells. In combination with the enhanced cell proliferation and invasion favored by the activation of EGF and MMP-9, TGF-β and VEGF also jointly promote angiogenesis and cell proliferation. ADAM10 a disintegrin and metalloprotease 10, AHR aryl hydrocarbon (or dioxin) receptor, AR androgen receptor, ARE androgen-responsive element, ARNT AHR nuclear translocator, ECM extracellular matrix, EGF epidermal growth factor, ERK extracellular signal-regulated kinase, GPER G-protein-coupled estrogen receptor, IL-6 interleukin 6, MAPK mitogen-activated protein kinase, MEK mitogen extracellular kinase, MICA/B major histocompatibility complex class I chain-related gene A/B, MMP-9 matrix metalloprotease 9, NK natural killer, PC pancreatic cancer, PSC pancreatic stellate cell, RAF Raf proto-oncogene, STAT3 signal transducer and activator of transcription 3, TAGs triglycerides, TAM tumor-associated macrophage, TCR T-cell receptor, TGF-β transforming growth factor β, TME tumor microenvironment, VEGF vascular endothelial growth factor. Panel **c** in this figure was adapted from a previous publication^195^

At first glance, the pancreas is certainly not one of the target organs of sex hormones. However, estrogen was found to be predominantly localized in the cytoplasm of acinar cells,^182^ which seems essential for the synthesis of pancreatic digestive enzymes. The increase in estrogen levels can also lead to elevated TAG and total lipid levels in the pancreas,^183,184^ suggesting that increased estrogen can contribute to fatty infiltration in the pancreas (Fig. 4). In contrast to promoting digestive enzyme synthesis, estrogen seems to have an inhibitory effect on pancreatic growth due to the reduced cell numbers.^185^ Moreover, estrogen treatment was shown to significantly suppress the progression of precancerous lesions in vivo.^186,187^

When discussing the role of estrogen in cancer, we must talk about the nuclear antagonist of estrogen receptors, the nonsteroidal drug tamoxifen, which has been used as standard endocrine therapy against breast cancer for decades. In addition to antagonizing estrogen receptors, tamoxifen also acts as an agonist of the G-protein-coupled estrogen receptor (GPER) expressed by many normal and malignant cells, commonly localized at intracellular membranes, regulating vascular tone and cell growth as well as lipid and glucose homeostasis. Hence, GPER is also implicated in obesity and DM.^188^ In terms of the roles of tamoxifen and GPER in PC, recent studies suggest that through the activation of GPER, tamoxifen reduces fibrosis and desmoplastic tissues by targeting PSCs and ameliorates the infiltration of macrophages by lowering the stiffness of the ECM while mitigating hypoxia and angiogenesis in the TME, which promotes apoptosis, inhibits cell proliferation, and prevents cancer progression^189,190^ (Fig. 4). Likewise, other agonists of GPER also showed satisfactory results in inhibiting cell proliferation and disrupting the cell cycle in PC.^191^ Together, these results indicate that GPER is a promising therapeutic target in the estrogen-related treatment of PC.

Androgen receptors (ARs) also exist in the normal pancreas and PC cells in humans.^192^ The overexpression of IL-6 in PC increases the phosphorylation of STAT3 and MAPK, which increases the activation of ARs in PC cells, promoting the progression of pancreatic carcinogenesis^193^ (Fig. 4). Furthermore, it was suggested that ARs might contribute to the progression of PC via the disruption of the circadian rhythm, a factor known to be associated with PC risk.^194^ Although it is assumed that ARs rather than androgen are involved in pancreatic carcinogenesis and progression,^195^ testosterone has been shown to vigorously promote experimental PC growth. In contrast, antiandrogen therapy has been shown to effectively prolong the survival of patients with unresectable PC.^185^

Based on the evidence above, along with the fact that men with obesity suffer from androgen deficiency with a mild increase in estrogen, while women with obesity have hyperandrogenemia and a prevalence of severe obesity approximately twice as high as that of men,^178^ it seems that women should be at greater risk of PC, which is obviously in contrast to the epidemiological data. Hypothetically, the best translation of these results would be, or likely be, the indirect role of sex hormones in exacerbating the abnormalities within the TME that contribute to metabolic dysfunction, inflammation, and carcinogenesis. Owing to these questions, more studies are warranted in the future to determine the roles of sex hormones in pancreatic carcinogenesis.

### Microbiomes

**Fig. 5:**
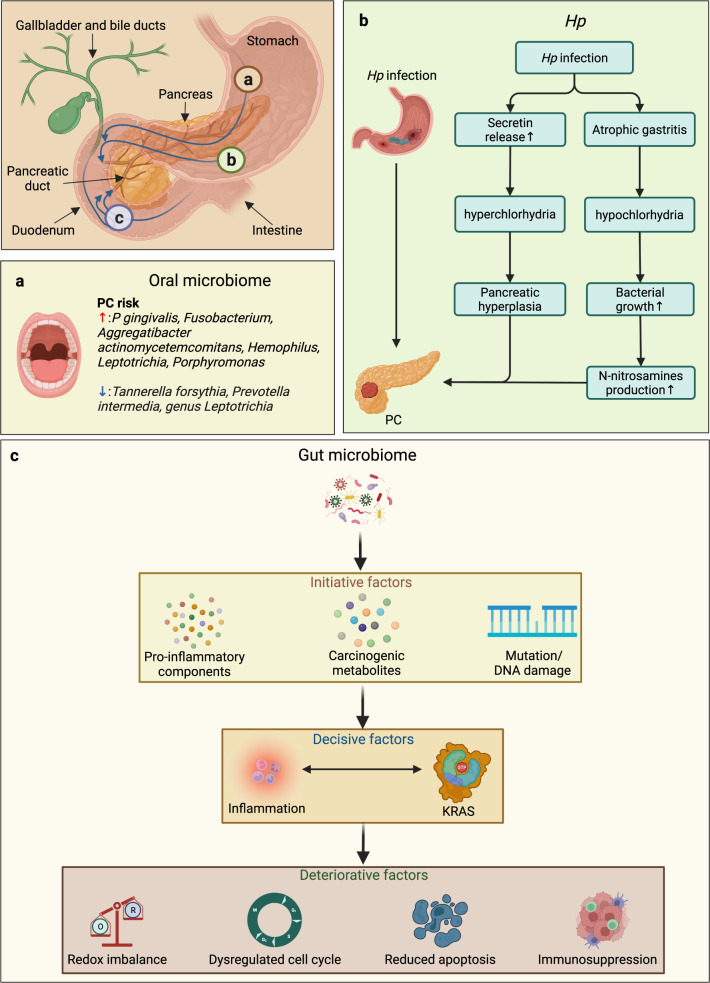
Microbes and pancreatic carcinogenesis. Upper left panel: Beyond their distant impact and transfer of their carcinogenic products, microbes from different regions of the GI tract may migrate to the pancreas via retrograde transfer through the opening of the sphincter of Oddi and contribute to pancreatic carcinogenesis. Marks a, b, and b (corresponding to panels **a**, **b**, and **c**, respectively) indicate the possible distant influence and transfer of oral, gastric, and GM carcinogenic products or their migration to the pancreas. **a** Distinct effect of different oral microbiome species on the risk of PC. **b** Two hypothetical theories on pancreatic carcinogenesis related to *Hp* infection. **c** Some metabolites of the GM, such as lipopolysaccharide (LPS), can enhance chronic inflammation by activating multiple carcinogenic pathways and increasing the secretion of proinflammatory components. In contrast, altered levels of carcinogenic metabolites (e.g., BAs) can promote pancreatic carcinogenesis by accelerating the senescence-associated secretory phenotype and increasing DNA damage and genomic instability. Some viruses from the GM community (e.g., HBV and HCV) are also suggested to increase inflammation-induced DNA damage and carcinogenesis. Later, microbe-induced inflammation can be carcinogenic and initiate the activation of KRAS, which also exacerbates inflammatory reactions in return. The enhancement of oncogenic signaling subsequently triggers other factors that promote the progression of carcinogenesis, such as oxidative stress, cell cycle disruption, suppressed apoptosis, and the immune response. BAs bile acids, GI gastrointestinal, GM gut microbiome, HB (C) V hepatitis B (C) virus, Hp *Helicobacter pylori*, KRAS Kirsten rat sarcoma viral oncogene homolog, PC pancreatic cancer

Microbiomes can interact with the immunometabolic and endocrine systems, and it has long been known that viruses and other microbes take part in carcinogenesis. Worldwide, nearly one-fifth of malignant conditions are associated with microbial infections,^196^ and this percentage might have increased in recent years owing to the rising prevalence of metabolic disorders and cancers. Emerging evidence suggests that changes in the diversity, composition, and dominant organisms of the microbiome are correlated with the occurrence and development of PC and impair chemosensitivity and antitumor immunity in patients with PC.^196^ Animal studies identified a time-dependent gut dysbiosis associated with KRAS activation in pancreatic tumors.^197^ Although most studies have focused only on the carcinogenic effects of the intestinal microbiome, microbes that inhabit other parts of the digestive tract are also indispensable and unneglectable, as they can all be carcinogenic in different ways (Fig. 5).

#### Oral microbiome

The oral cavity contains a vast diversity of bacteria, viruses, and fungi,^198^ and these commensal microbes can be pathogenic and even carcinogenic under certain conditions.^199^ Periodontal disease, for example, an inflammation caused by oral microbes, has been suggested to elevate the risk of PC^200^ owing to alterations in microbial composition^201^ and the immune response.^200,202^ Specifically, high amounts of *Porphyromonas gingivalis* were shown to increase the PC risk up to 2-fold, while high levels of antibodies against nonpathogenic oral bacteria were shown to reduce this risk.^203^ Likewise, carriers of *P. gingivalis* share the same higher risk, regardless of the abundance of the bacteria, which indicates that *P. gingivalis* may serve as a biomarker for PC screening.^203^ Moreover, some periodontal *Fusobacterium* species were also detected in PC samples, but their roles remain elusive. In contrast to the increased risk of PC due to the carriage of the periodontal pathogen *Aggregatibacter actinomycetemcomitans*, *Tannerella forsythia, Prevotella intermedia*, and *the genus Leptotrichia* are associated with a decreased risk^203^ (Fig. 5). Of note, *Fusobacterium, Haemophilus, Leptotrichia*, and *Porphyromonas* are also suggested to be sensitive in distinguishing patients with PC.^203^ Mechanistically, the oral microbiome can promote pancreatic carcinogenesis by migrating to the pancreas through the natural digestive tract or the circulation during bacteremia, disrupting the pancreatic microenvironment.^200^ One of the most studied oral microbes, *P. gingivalis*, was speculated to increase *p53* and *Kras* mutations following degradation through peptidyl-arginine deiminase enzyme secretion.^204^

##### Helicobacter pylori

As the only bacterium colonizing the stomach, the relationship between *Helicobacter pylori* (*Hp*) and gastric cancer has been recognized previously. Other studies in recent years have also connected *Hp* with PC.^200^ Accordingly, PC patients are more likely to test positive for *Hp*.^205^ However, there is no widely accepted explanation for the causality of *Hp* in pancreatic carcinogenesis. Some have proposed that pancreatic hyperplasia resulting from hyperchlorhydria and excessive release of secretin following *Hp* infection could be a possible answer, whereas others argued that atrophic gastritis and hypochlorhydria causing bacterial overgrowth and N-nitrosamine overproduction are the culprits^200^ (Fig. 5). Both of these findings coincide with the theory that the carcinogenic effects of *Hp* lie in chronic mucosal inflammation and aberrant cell proliferation and differentiation.^206^

#### Pancreatic microbiome

Contradictory to the obsolete views that the pancreas is sterile, previous findings proved that not only does the pancreas have its own microbiome, but there are also considerable differences in microbe abundance and composition between normal and cancerous pancreatic tissue.^207^ A previous study analyzing the microbes in pancreatic cyst fluid illustrated their unique microbial ecosystem and detrimental influence on the pancreatic neoplastic process, with this correlation being found with microbiome composition rather than microbe abundance.^208^ Later, the discovery of bile transporting some gut microbes to the pancreas^209^ further demonstrated the direct contact with and impact of the GM on the pancreatic microenvironment.^207^

#### The gut microbiome

Influenced by age, dietary habits, antibiotics, and other internal and external environmental factors, the GM is the most studied microbiome with a crucial impact on obesity, DM, and carcinogenesis^210^ (Fig. 5). However, only a few species are recognized as being carcinogenic due to their extensive colonization of the GI tract, manifesting vast complex interactions among the GM, environmental factors, and cancer initiation.^211^ Undoubtedly, obesity, DM, and carcinogenesis are subsequent chain reactions resulting from dysbiosis and the subsequent generation of toxic metabolites.^212^ As mentioned above, GM dysfunction leads to alterations in the levels of GI hormones, glucose hemostasis and energy balance. Moreover, some metabolites, such as LPS, enhance chronic inflammation, while BAs promote carcinogenesis by accelerating the senescence-associated secretory phenotype, increasing DNA damage and genomic instability and activating carcinogenic signaling pathways or inducing direct tumorigenic effects.^211^ For instance, *Fusobacteria* was shown to provoke NF-κB signaling and increase the production of proinflammatory cytokines, such as IL-1, IL-6, IL-8, TNF-α, MMP-3, and COX2.^173^

#### Fungi and viruses

Less-studied fungi and viruses are also associated with pancreatic carcinogenesis. While clinical trials in distinct populations found that *Candida* infection increases the risk of PC,^200^ preclinical research showed exponential growth of pathogenic fungi and altered composition of the mycobiome in PDAC in both humans and rodents, which promote disease development by driving the complement cascade with the cleavage of C3 into C3a and C3b through the activation of mannose-binding lectin (MBL).^213^ Notably, intrapancreatic fungi can increase more than 3000 times in number in PDAC compared with the normal pancreas, and the latest study also demonstrated that translocated fungi are capable of augmenting the production of IL-33 from PDAC cells to enhance the recruitment and activation of immunosuppressive T helper 2 (T_H_2) cells and type 2 innate lymphoid cells (ILC2s), thereby promoting the progression of PDAC.^214^ In addition, hepatitis viruses B and C (HBV and HCV, respectively) are also suggested to be associated with PC through inflammation-induced DNA damage and carcinogenesis.^200,203^

#### The main mechanisms by which microbes promote pancreatic carcinogenesis

##### Systemic and pancreatic inflammation

Microbial infections can cause carcinogenic inflammation in the pancreas, whether locally or systemically, since the constant stimulation of inflammation driven by the microbes was suggested to initiate the activation of KRAS,^215^ which also involves several other cancer-related inflammatory signaling pathways.

First, macropinocytosis is regulated by Wingless/Integrated (Wnt) signaling. Macropinocytosis is an endocytic process of antigen capture, presentation, and subsequent activation of the inflammatory reaction.^216^ Wnt signaling mediates the proliferation and differentiation of cells and tumor growth during pancreatic carcinogenesis.^200^ Consistently, a previous study demonstrated that fat mass and obesity-associated protein (FTO), an essential regulator of obesogenesis, mitigated pancreatic carcinogenesis by demethylating PJA2 and diminishing Wnt signaling.^217^ The next step is the stimulation of TLRs by LPS. TLR4 activates several downstream pathways that are carcinogenic in inflammatory conditions. mTOR signaling, for example, not only reshapes the composition of the GM but also participates in pancreatic carcinogenesis by promoting tumor growth through ERK/mTOR signaling.^200^ In addition, the interactions between LPS and TLR4 can activate NF-κB and STAT3 signaling and accelerate carcinogenesis by amplifying RAS signals and enhancing the progression of tumors.^200,211,218^ Similarly, the increased levels of inflammatory cytokines in obesity and DM, such as IL-1, IL-6, and TNF-α, also participate in the carcinogenic process via the activation of the NF-κB pathway.^219^ Furthermore, the activation of other pathways, such as the JNK/AKT/STAT3 and cyclin/cyclin-dependent kinase (CDK) pathways, increases oxidative stress, disrupts the cell cycle, suppresses apoptosis, and induces DNA mutations.^203^

Apart from the inflammation- and damage-induced metaplasia resulting from HBV and HCV infection in the pancreas, these two viruses can also promote carcinogenesis by causing a high level of mutations in the *TP53* and *CTNNB1* genes, activating numerous oncogenic processes, including telomere maintenance, Wnt signaling, cell cycle regulation, oxidative stress, epigenetic modifications, JAK/STAT signaling, immune response suppression and apoptosis.^203^

##### Diminished immune response

Interacting bidirectionally with each other, the microbiome and the immune system collaboratively maintain the symbiosis between the human body and microorganisms, while the immune system influences the composition and evolution of the microbiome, which in turn affects the maturation and adaptation of the immune system as well as the carcinogenesis caused by immune dysregulation.^211^ Different studies have emphasized the two almost opposite effects of the microbiome: the promotion of immune maturation and the suppression of antitumor immunity.

On the one hand, the gut microbiome and the immune system can affect each other in the gut lamina propria and extraintestinal sites. Microbes can act as antigens activating the immune system to promote its maturation and maintain its functional integrity.^200^ Some specific species, such as *Bacteroides fragilis* and *Bifidobacterium* species, were deemed essential for the maturation of the immune system.^200^ On the other hand, microbe-mediated immune suppression is associated with pattern recognition receptors (PRRs), which are also called pathogen-associated molecular patterns (PAMPs) and are capable of directly recognizing pathogens of microorganisms. The TLR and nucleotide-binding oligomerization domain (NOD)-like receptor (NLR) families are two major sets of receptors that induce carcinogenic effects in GM-related inflammation.^211^ As introduced, TLRs can recognize microbial pathogens (e.g., LPS, lipoproteins, lipopeptides, flagellin, single- or double-stranded DNA, and CpG DNA) and trigger the inflammatory response and carcinogenesis.^211^ In addition, the activation of the NF-κB and MAPK signaling pathways following the stimulation of TLRs initiates the production of proinflammatory cytokines and the recruitment of inflammatory entities, accelerating the development of cancer.^220^ NLRs, likewise, can promote carcinogenesis by increasing the release of inflammasomes and ILs after recognizing microbial signals while activating NF-κB, p38 MAPK, and interferon signaling to modulate bacterial clearance and augment the formation of autophagosomes.^211^ The inhibition of these receptors diminishes tumor development, whereas several other TLRs (e.g., TLR2, TLR4, TLR5, and TLR7) suppress innate and adaptive immunity to promote the development of PC by disrupting the interactions between macrophages and lymphocytes.^200^

Different microbiomes play distinct roles in immunity or other tumor models. With advancing techniques in sample analysis and sequencing [e.g., single-cell analysis of host-microbiome interactions (SAHMI)^221^], the future combination of these methods with other multiomic data will reveal an increasing number of roles of the microbiomes in immune dysfunction in pancreatic carcinogenesis resulting from metabolic disorders.

##### Changes in metabolism

As mentioned above, microbes are one of the main regulators of energy balance. Given that obesity is characterized by altered microbial diversity, the excessive release of LPS from the GM in obesity often leads to endotoxemia, with this low-grade chronic inflammation increasing the risk of PC by augmenting the secretion of various proinflammatory cytokines and activating the NF-κB pathway.^200^ Regarding the roles of the microbiome in DM, in addition to the insulin resistance caused by GM dysfunction, it was suggested that alterations in the levels of metabolites, such as acetate and butyrate, also increase the risk of PC by enhancing chronic inflammation through endotoxemia due to impaired epithelial tight junctions in the intestinal mucosa.^200^

### The TME and cellular perturbations

The TME comprises infiltrating immune cells, such as lymphocytes, TAMs, mast cells, antigen-presenting cells (APCs), and granulocytes, as well as CAFs, endothelial cells, ECM, and other stromal components.^222^ As the hallmark and most frequently mutated oncogene in PC, mutated *KRAS* cooperates with existing metabolic abnormalities to further influence the different components of the pancreatic TME.^223^ Mutated *TP53*, another commonly mutated gene, has been suggested to deteriorate fibrosis and immunosuppression within the TME of PDAC.^224^ TME aberrance results in epithelial dysfunction, carcinogenesis, and tumor promotion. Specifically, the ectopic expansion of adipose tissue fuels energy imbalance and inflammatory disruption in the TME through excessive production of proinflammatory chemokines and cytokines and dysregulated secretion of adipokines. Meanwhile, cancer-associated adipocytes (CAAs) further scatter the TME and provide crucial support for the progression of carcinogenesis, with hyperactive CAFs, ECM deposition, and hypoxia promoting fibroinflammatory desmoplastic reaction, EMT, and immunosuppression to promote tumor formation.

#### Ectopic adipose tissue expansion and the adipose tissue microenvironment

##### Adipose tissue microenvironment

**Fig. 6:**
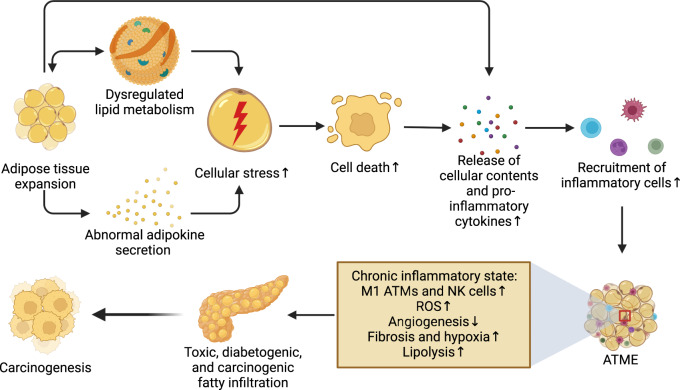
Correlations between the ATME and pancreatic carcinogenesis. Beyond an immediate increase in the development of low-grade chronic inflammation via the enhanced secretion of proinflammatory cytokines, the excessively expanded adipose tissue exacerbates metabolic disorders and magnifies the negative impact of adipokines. Meanwhile, this hypertrophic expansion inflicts stress on adipocytes and increases the production and release of substantial proinflammatory cytokines into the ATME, promoting the recruitment and proliferation of inflammatory cells and exacerbating oxidative stress, fibrosis, hypoxia, and lipolysis, which collaboratively have toxic, diabetogenic, and carcinogenic influences on the pancreas. ATMs adipose tissue macrophages, ATME adipose tissue microenvironment, NK natural killer, ROS reactive oxygen species

In obesity, the excessively expanded adipose tissue, including subcutaneous and visceral adipose tissue and the adipose tissue surrounding many organs, is a vital organic system capable of modulating the production of adipokines, inflammatory cytokines, and other enzymes that potentially contribute to carcinogenesis and tumor growth while exerting an essential impact on cancer cells in the adipose tissue microenvironment (ATME). Weight gain is accompanied by anti-inflammatory to proinflammatory status elevation in the ATME owing to hypertrophy and adipocyte death, increasing the production and release of multiple proinflammatory cytokines into the ATME [e.g., TNF-α, interferon γ (IFN-γ), IL-1β, and IL-6)]^225^ and thus exacerbating chronic fibrosis and vascular inflammation, which in turn disrupts ATME homeostasis^226^ (Fig. 6). Specifically, the different types of adipocyte death in obesity result in the release of cellular contents such as lipids, cytokines, and other signaling molecules into the ATME, promoting the recruitment and proliferation of phagocytic macrophages.^227,228^ Macrophages scavenge lipids and cellular debris by encircling dying adipocytes and forming crown-like structures (CLSs) and sometimes evolve into other cell types.^229^ However, the direct outcomes of these processes are simple: the activation of several inflammatory pathways, including the NLR and TLR pathways, and downstream signaling through inflammasome activation, an inhibitor of NF-κB kinase subunit β (IKKβ)-NF-κB 47 and c-JNK1 (also known as MAPK8), which are all correlated with insulin resistance and metabolic abnormalities in obesity and DM. Hence, the formation of CLSs in the ATME is regarded as a principal lesion and biomarker of adipose tissue inflammation,^228^ which is also implicated in obesity-induced DM.^230^

To date, most of the evidence regarding the roles of CLSs in cancer is based on cancer types other than PC, such as breast cancer. CLSs are more frequently detected among obese than nonobese breast cancer patients, are associated with an increased risk of breast cancer and contribute to the development and progression of cancer as metabolic and inflammatory factors.^231^ Similarly, in prostate cancer, CLSs and concurrent inflammation in periprostatic fat were shown to be associated with a higher body mass index (BMI) and tumor grade in men,^232^ and the results from rodent studies suggest that supplementation with estrogen and caloric restriction could be an ideal anti-inflammatory therapeutic option in the treatment of obesity.^233^ In the context of the consistent results from studies of other nonhormone-driven cancers^234^ and the connections between inflammation and pancreatic carcinogenesis in obesity and DM, we can assume that CLSs could also be essential drivers of pancreatic carcinogenesis, and further studies are warranted for confirmation.

##### Fatty infiltration in the pancreas

Ectopic visceral adipose tissue synthesizes various adipocytokines involved in metabolic processes, inflammation, appetite regulation, immunity, hematopoiesis, angiogenesis, and diseases such as obesity and cancer.^235,236^ Obesity causes intrapancreatic fatty infiltration associated with PanIN.^174^ Consistently, previous studies have indicated that intrapancreatic adipose tissue could be toxic, diabetogenic^237^ and carcinogenic^238^ since adipocytes are also endocrinologically capable of producing many molecules, including hormones, GFs, and adipokines, to reshape the local environment,^239,240^ making it conducive the progression of PanIN and consequent PC development (Fig. 6).^241^

With the gradual increase in pancreatic fat until the age of 60 years and the slow decrease in the volume of the pancreatic parenchyma after the age of 30 years, the fat/parenchymal ratio increases with age and results in fatty infiltration in the pancreas.^242^ When combined with metabolic dysregulation, such as the dyslipidemia and excessive visceral adipose accumulation in obesity and T2DM, fatty infiltration can be even more severe, and it is positively associated with the risk of PC.^243^ Unlike in hepatosteatosis, human samples have demonstrated that adipocytes infiltrate the pancreatic parenchyma in a scattered pattern (intralobular fat) and accumulate in the perilobular space, mainly around large vessels (interlobular fat).^244^ While intralobular fat is speculated to be produced by transforming fibroblasts or acinar cells to fill in the spaces created by the loss of damaged acinar cells, interlobular fat is seemingly related to obesity and T2DM.^244^ Clinical sample analysis demonstrated that fatty infiltration is more common in the peritumoral tissues of PC patients, where the infiltration fraction correlates with BMI and HbA1c levels in both groups.^241^ Even in the precancerous stage, pancreatic fatty infiltration correlates with the progression of PanINs in obesity.^245^ The potential mechanisms of these carcinogenic processes include the reprogramming and remodeling of the immunosuppressive TME, abnormalities in inflammation via the aberrant secretion of proinflammatory cytokines, and the disruption of growth factor signals (which will be introduced in detail below).

#### White adipose tissue inflammation

Generally, adipose tissue in patients with obesity is characterized by hypertrophy, hyperplasia, and an increased number of preadipocytes.^246^ Preadipocytes, by producing inflammatory cytokines and chemokines, as well as attracting and activating macrophages and endothelial precursors, create a proinflammatory microenvironment by increasing the levels of proinflammatory cytokines such as leptin, TNF-α, retinol-binding protein 4 (RBP-4), VEGF, IL-6, IL-8, IL-17, C-C motif chemokine ligand (CCL) 2, and CCL5,^247,248^ while infiltrated macrophages produce IL-6, IL-8, CCL2, and CCL5 at the same time.^247–249^ As a result, the established inflammatory microenvironment supports the proliferation of preadipocytes but impairs their differentiation.^250^

The excessively accumulated and expanded white adipose tissue (WAT) in patients with obesity is infiltrated by immune cells (mostly macrophages and lymphocytes), which secrete proinflammatory mediators to foster tumor growth.^251^ In addition, the expanded WAT outgrows its blood supply, leading to hypoxia, which causes adipocyte stress and death.^252^ Apart from the proinflammatory mediators secreted by enlarged adipocytes, the FFAs released from adipocytes and other cells jointly activate TLR4 in macrophages, leading to the increased expression of proinflammatory genes dependent on NF-κB, such as *TNFα*, *IL1β*, and *COX2*.^253^ Conversely, TNF-α and other cytokines sustain WAT inflammation by stimulating lipolysis and the release of FFAs.

The impact of adipose tissue dysfunction extends far beyond the TME; adipose tissue dysfunction also elicits a systemic effect that may synergistically fuel tumor growth. WAT inflammation is also associated with elevated circulating levels of various adipokines, C-reactive protein (CRP), and IL-6 in patients with obesity and DM, which have all been shown to promote pancreatic carcinogenesis.^254–256^ Therefore, it is likely that both the local and systemic environments are reprogrammed to promote carcinogenesis under conditions of WAT dysfunction and inflammation.

##### Adipokines

Adipokines are secreted by adipocytes, while cytokines are mainly produced by immune cells infiltrating adipose tissues, including macrophages and lymphocytes. Many studies have revealed the multifaceted roles of these signaling molecules in obesity- and DM-related carcinogenesis, and some of the most studied adipokines and cytokines will be addressed below.

1. *Adiponectin*. The most abundant adipokine in circulation, adiponectin, was also reported to be the first dysregulated hormone in metabolic disorders.^257^ Although adiponectin is primarily produced by adipose tissues, the levels of circulating adiponectin are inversely decreased in patients with obesity and DM. Adiponectin functions as an insulin-sensitizing, antidiabetic, anti-inflammatory, antiangiogenic, and anticancer adipokine. Adiponectin abolition in mice resulted in increased expression of proinflammatory genes, whereas adiponectin treatment reversed these effects. Moreover, adiponectin participates in the maturation of preadipocytes,^258^ and its signaling increases the phosphorylation of AMP-activated protein kinase (AMPK) and antagonizes leptin signaling.^259^
  Previous research indicated that hypoadiponectinemia is associated with a higher risk of PC.^260,261^ Consistently, adiponectin has been shown to restrain tumor development and growth by affecting several intracellular signaling pathways, including the MAPK, mTOR, PI3K/AKT, STAT3, NF-κB, and sphingolipid metabolic pathways.^262,263^ Adiponectin also significantly inhibits the proliferation of PC cells by blocking the phosphorylation/inactivation of GSK-3β and suppressing the intracellular accumulation of β-catenin, reducing the expression of cyclin D1 and causing cell cycle arrest.^264^ Adiponectin has been shown to have indirect antitumor activity through its insulin-sensitizing and anti-inflammatory effects as an antineoplastic therapy,^265^ and it has been deemed to be an ideal diagnostic and prognostic biomarker in cancer.^257^ Nevertheless, a Mendelian randomization study with a large sample size did not find any correlation between circulating adiponectin or leptin and PC,^266^ and one study even suggested that adiponectin could be cancer-promoting in a tissue-dependent manner.^267^ Therefore, the roles of adiponectin in obesity- and DM-related pancreatic carcinogenesis are still controversial.
2. *Leptin*. Leptin is an adipokine involved in the regulation of energy balance, and its receptors are widely expressed in peripheral tissues, including pancreatic β-cells,^268^ with their activation reducing insulin secretion and modulating the proliferation, apoptosis, and size of β-cells.^269^ Notably, the adipogenic insulin overproduced in patients with obesity and T2DM stimulates the production and secretion of leptin, which in turn suppresses insulin secretion through both a central effect and its direct action on β-cells, establishing a hormonal regulatory feedback loop, a so-called adipo-insular axis.^270^ In addition, an elevated level of leptin is common in individuals with obesity and DM due to leptin resistance without any reduction in appetite.^248^ In contrast to adiponectin, which has antiproliferative and anti-inflammatory functions, leptin has mitogenic and proinflammatory effects by stimulating the production of IL-1, IL-6, IL-12, TNF-α, leukotriene B4 (LTB-4), and COX2.^271^ Leptin is also involved in the differentiation of monocytes and promotes T-cell proliferation and the T_H_1 phenotype while suppressing regulatory T cells (Tregs).^271^
  Consistent with the evidence mentioned above, an increased leptin level is correlated with PC risk.^255,272^ However, while some researchers have also proposed a correlation between decreased leptin levels and PC,^273^ none of these correlations were reported in a previous Mendelian randomization study.^266^ By binding to its full-length receptor, leptin activates numerous intracellular signaling pathways that promote cancer cell growth and PC progression, including the leptin-Notch/RBP/JNK, JAK/STAT, MAPK, PI3K/AKT, and suppression of cytokine signaling (SOCS) pathways.^274–276^ Additionally, classic histone deacetylase (HDAC)-microRNA-leptin signaling can be oncogenic and increase proliferation, cellular stemness, and angiogenesis in PC.^277^ For further details, the roles of leptin in carcinogenesis have been reviewed elsewhere.^278^
3. *Lipocalin-2 (LCN2)*. LCN2, also known as neutrophil gelatinase-associated lipocalin (NGAL), siderocalin, or 24p3, belongs to the lipocalin superfamily as a pleiotropic mediator of various inflammatory processes.^279^ Consistently, circulating levels of LCN2 are increased in patients with obesity, insulin resistance, and DM, with a parallel elevation in inflammatory markers, such as TNF-α, IL-1β, IL-6, and IFN-γ, exerting a proinflammatory effect.^280^ Since LCN2 can be secreted by adipocytes, neutrophils, macrophages, and cancer cells, elevated levels of LCN2 can be detected in obesity, DM, and PC.^281–283^ Overall, the increased level of LCN2 is correlated with carcinogenesis and progression to PC, in which LC2 levels begin to rise early in PanIN development and increase constantly during malignant progression to PDAC.^284^ Although the changes in the levels of LCN2 in animals led to inconclusive findings,^284^ the elevated level of LCN2 has been shown to contribute to the establishment of an inflammatory TME in PC via increased secretion of proinflammatory cytokines, such as IL-1β, IL-6, IL-8, stromal cell-derived factor 1 (SDF-1), intercellular adhesion molecule 1 (ICAM-1), MMP-1, MMP-3, MMP-9, and α-smooth muscle actin (α-SMA) from PSCs,^281,285,286^ which exacerbates the infiltration of TAMs^285^ and collaboratively promotes pancreatic carcinogenesis. In addition to its roles in modulating the TME, the elevation of LCN2 was also reported to be an early biomarker of PDAC in human samples of urine, serum, bile, pancreatic fluid/juice, and pancreatic cyst fluid.^284^ However, since there are also speculations about LCN2 being a tumor suppressor gene depending on the specific type of cancer, LCN2 is of great interest for further investigations.
4. *Resistin*. Resistin is primarily secreted by macrophages via the induction of IL-1, IL-6, and TNF-α. Similar to leptin, resistin is a proinflammatory adipokine responsible for insulin resistance in obesity and T2DM through SOCS3.^287^ Given that increased levels of resistin are associated with PC risk,^288^ it was reported that resistin activates STAT3 in an adenylyl cyclase-associated protein 1 (CAP1)- and TLR4-dependent manner to support the proliferation of PC cells and thereby contribute to tumor progression.^289^ In addition, resistin functions as a regulator of inflammation in the signaling of general transcription factor II-I repeat domain-containing protein 1 (GTF2IRD1), promoting the progression of PC.^290^
5. *Nicotinamide phosphoribosyltransferase* (NAMPT)*/visfatin*. NAMPT is secreted by adipose tissue and many other tissues in humans, acting as an enzyme, adipocytokine, and growth factor.^236^ Among the two forms of NAMPT, intracellular NAMPT (iNAMPT) participates in nicotinamide adenine dinucleotide (NAD) biosynthesis, a crucial part of cellular metabolism. The other form, extracellular NAMPT (eNAMPT), is implicated in several pathologies.^236^ In detail, circulating levels of NAMPT are increased in patients with obesity and T2DM,^291^ and NAMPT was also demonstrated to increase the expression of proinflammatory cytokines and inhibit apoptosis in response to endoplasmic reticulum (ER) stress.^291^ Additionally, eNAMPT exerts proinflammatory, proproliferative, antiapoptotic, and proangiogenic effects through the activation of the NF-κB, Notch-1, cyclin D1, CDK 2, MAPK, ERK1/2, and p38 signaling pathways.^236^ Furthermore, NAMPT is the rate-limiting enzyme of the NAD salvage pathway that is vital for the proliferation and survival of PC cells.^292^ Hence, inhibiting the NAD salvage pathway often causes metabolic collapse and cell death. In addition to reduced viability and colony formation in different PDAC cell lines, specific NAMPT inhibitors also decreased glucose uptake, lactate excretion, and ATP levels, resulting in metabolic collapse through activated AMPK and inhibited mTOR pathways.^293^ Likewise, a clinical trial also validated the efficacy of NAMPT inhibition.^294^ Given the roles of NAMPT in cellular metabolism and that IL-1 can stimulate the production of NAMPT,^295^ it is likely that inflammatory conditions in obesity and DM increase the levels of NAMPT initially and contribute to pancreatic carcinogenesis by promoting glycolysis and inflammation.
6. *Chemerin and omentin-1*. Expressed in multiple sites in humans, chemerin and omentin-1 (also known as intelectin-1) are both produced by adipose tissue and related to metabolic disorders in obesity and DM. Chemerin is involved in innate and adaptive immunity, adipogenesis, and adipocyte metabolism, while omentin-1 exerts its various functions in the regulation of metabolic processes in endocrine, autocrine, and paracrine manners.^236^ The levels of chemerin and omentin-1 are similarly increased in patients with PC, with the former showing a better discriminant ability.^296^ Chemerin can promote carcinogenesis through the inflammation, angiogenesis, and recruitment of mesenchymal stromal cells (MSCs),^296,297^ while omentin-1 can also promote cancer cell proliferation via enhanced inflammation.^297,298^ However, the effects of chemerin and omentin-1 differ among cancers.
7. *Retinol-binding protein 4 (RBP-4)*. RBP-4 is an adipocytokine that acts by binding to cell surface receptors or through retinoic acid and retinoic acid-X receptors and is associated with TAG, total cholesterol, and LDL-cholesterol dysregulation and high blood pressure.^236^ Although some have questioned the diagnostic specificity of RBP4 in PC,^299^ others have shown that increased RBP-4 is effective in discriminating PC in patients with T2DM.^300^ Consistently, RBP-4 was suggested to promote JAK/STAT signaling via its receptor stimulated by retinoic acid 6 (STRA6) in carcinogenesis.^236^
8. *Osteopontin (OPN)*. OPN, a protein of the bone calcified matrix, is a proinflammatory adipocytokine expressed in different cell types^301^ and is involved in biomineralization, remodeling and metabolic disorders such as obesity and DM.^302^ In cancer, OPN impacts cell proliferation, survival, drug resistance, invasion, and cell stemness by mediating cellular crosstalk and influencing the TME.^303,304^ OPN was shown to be tumorigenic a decade ago,^305^ and a higher OPN level is a diagnostic biomarker of PC.^306,307^ Concerning the roles of OPN isoforms in obesity, DM, and PC, OPNc was closely associated with obesity and DM, whereas the overexpression of OPNb and OPNc in PDAC cells increased the formation of colonies and the transcription of *IL6*.^308^ In addition, in a cell model replicating DM-related PC, the increased oxidative stress fueled by high glucose levels was reported to accelerate cell proliferation and mRNA expression of OPN in human pancreatic duct epithelial cells.^309^ Similarly, in a ROS-dependent manner, OPN secreted from activated PSCs in hypoxia interacts with the transmembrane receptor integrin αvβ3 on PC cells to upregulate the expression of forkhead box protein M1 (FOXM1), which induces malignant phenotypes of PC cells by enhancing EMT and cancer stem cell (CSC)-like properties through the AKT and ERK pathways.^310^ Upregulated OPN also takes part in the subtype conversion from classic to basal-like in PDAC through the modulation of the transcription factor *GLI family zinc finger 2* (*GLI2*), which is a driver of PDAC cell plasticity that is critical for metastatic growth and adaptation to oncogenic *KRAS* ablation.^311^ Beyond that, OPN induces the activation of MMP-2 and MMP-9,^236^ which are known to promote carcinogenesis in PDAC.^312^
9. *Oncostatin M (OSM)*. As a part of the inflammatory response in obesity and DM, OSM belongs to the IL-6 family and is secreted by activated T cells and macrophages in WAT.^313^ Despite being initially introduced as an anticancer agent, OSM can promote carcinogenesis and cancer progression in some cases, and the overexpression of OSM and OSM receptor (OSMR) has been detected in various cancers, including PC.^314^ OSM is elevated in the serum of PDAC patients,^315^ with TAMs in murine PDAC exerting increased secretion of OSM as well.^316^ OSM activates several carcinogenic signaling pathways through the formation of a complex composed of OSM and the cell signaling molecule gp130, including JAK/STAT3, MAPK, and PI3K.^317^ Among them, STAT3 is the main downstream signaling molecule of OSM regulating cell growth, invasion, survival, and all other hallmarks of cancer cells.^314^ In line with its unique efficacy compared to other members of the IL-6 family, OSM was shown to be one of the strongest drivers of EMT and CSC plasticity fueled by the induction of ZEB1, Snail (SNAI1), and OSMR, leading to enhanced pancreatic tumorigenicity through the JAK/STAT3 pathway.^318,319^ In addition, the cooperative signaling among OSM, hepatocyte growth factor (HGF), and TGF-β improves EMT in PC.^320^

In summary, adipocytes support tumor metabolism, and adipocytokines mediate carcinogenesis and cancer progression via their paracrine and endocrine actions. In obesity and DM, adipocytokines present independent and joint effects on the activation of major intracellular signaling pathways implicated in the carcinogenesis, proliferation, expansion, and survival of cancer cells. Treatments targeting obesity and DM have been shown to alter the plasma levels of adipocytokines and influence cancer risk. Therefore, many studies are being conducted to validate the potential of adipokines as therapeutic targets for the treatment of obesity and DM and diagnostic biomarkers of cancers. For now, more translational research is essential considering the conflicting results of the roles of some adipokines (e.g., adiponectin and leptin) in pancreatic carcinogenesis.

#### Cancer-associated adipocytes

Peritumoral adipocytes that are capable of interacting with cancer cells and contributing to tumor formation and growth by providing energy sources are called CAAs.^251^ The crosstalk between CAAs and cancer cells is important in determining the biological behavior of a tumor. Characterized by a strange fibroblast morphology, smaller and scattered lipid droplets, enhanced active energy metabolism and secretion, and higher expression of adipokines and chemokines, the concept of CAAs was proposed in 2010.^321^ Adjacent adipocytes also undergo similar phenotypic changes orchestrated with the remodeling of the TME. Currently, it is widely accepted that adipocytes in the inflammatory TME can dedifferentiate into CAAs that are fibroblast-like and capable of promoting ECM remodeling, tumor growth, and tumor progression.^322^ Meanwhile, PC cells can induce the dedifferentiation of adjacent adipocytes to CAAs, reducing their lipid droplets and significantly enhancing the EMT, migration/invasion capability, and chemoresistance of cancer cells.^323^ In addition to the pancreatic fatty infiltration mentioned above, enhanced Wnt5α signaling, a noncanonical Wnt pathway, plays a key role in the dedifferentiation of adipocytes in PC by favoring an inflammatory TME via the production of IL-6 and STAT3.^324^ In addition to the effects of adipocytes promoting carcinogenesis via adipokine and chemokine secretion, CAAs support pancreatic carcinogenesis via remodeled metabolism and crosstalk with other cells within the TME.

Cancer cells must rewire their metabolism to survive in a hypoxic TME due to fibrosis, the lack of vasculature, and poor perfusion. Thus, reprogramming lipid metabolism would be an ideal solution. Lipid metabolism is upregulated in the hypoxic TME.^325^ Apart from synthesizing FAs on their own, cancer cells also take up lipoproteins, chyme particles, and FFAs from adipocytes to sustain their constant proliferation.^326^ Unlike the storage of lipid droplets in hepatocytes in the liver, the lipids in the pancreas are mainly stored in infiltrated adipocytes in a much smaller amount,^327,328^ implying the potential active lipid supply from adipocytes to PC cells. However, it was also confirmed that exosomes secreted by PC cells increase lipolysis in adipose tissue.^329^ As a result, elevated FAs resulting from lipolysis act as a powerful driver of carcinogenesis, as addressed above.

As introduced in the last section, adipocytes can produce a wide range of adipokines and chemokines to promote carcinogenesis. These signaling molecules enable crosstalk between CAAs and other cell types in the TME, such as PSCs, tumor-associated neutrophils (TANs), Tregs, and CD8^+^ T cells, to fuel immunosuppression as well.^246,330^ Therefore, targeting CAAs has now been deemed a therapeutic strategy against PC. As a vital part of the TME, the roles of adipocytes in carcinogenesis are worth further investigation, which would be key to dissecting the impacts of adipose tissue on obesity- and DM-related pancreatic carcinogenesis.

#### CAFs, CAF-induced immunosuppression, disrupted ECM deposition, and hypoxia

##### CAFs

As a solid tumor, PC is characterized by a fibrotic and highly desmoplastic TME, highlighting the extensive distribution and significance of fibroblasts and PSCs in its pathogenesis. Resident fibroblasts and PSCs maintain the connective tissue architecture of the normal pancreas. Nevertheless, in collaboration with CAFs, PSCs induce excessive production of inflammatory cytokines and tissue-healing proangiogenic GFs that enhance the recruitment of both adaptive and innate immune cells, angiogenesis, and the deposition of ECM in chronic inflammation.^331^ Likewise, a recent study has also demonstrated that DM can activate PSCs through RAGEs in PDAC.^332^ Later, following the transformation of PSCs induced by various GFs and cytokines, such as pigment epithelium-derived factor (PEDF), endothelin 1 (ET-1), IGF-1, trefoil factor 1 (TFF2), and platelet‐derived growth factors (PDGFs), both PSCs and CAFs can change their phenotypes, resulting in excessive secretion of ECM during carcinogenesis, promoting cancer cell proliferation and reducing apoptosis.^331^ Although more than ten types of cells have been recognized as precursors of CAFs, there are only three well-defined subtypes of CAFs in PDAC, with considerable biological heterogeneity in spatial distribution and functional properties rooted in their diverse cell of origin: 1) profibrotic, immunosuppressive, and tumor restraining myofibroblastic CAFs (myCAFs); 2) secretory and inflammatory CAFs (iCAFs), which are functionally similar to senescent fibroblasts and deemed to be immunosuppressive and tumorigenic; and 3) immunoregulatory and tumorigenic antigen-presenting CAFs (apCAFs).^333^

Regardless of the differences in methods and standards used in discriminating the subtypes of CAFs, the correlations between their distinct features and PC are definite.^334^ Moreover, since each subtype has specific markers, combining subtype-specific lineage tracing models and in vivo imaging methods is ideal for revealing the cellular origin of distinct CAF populations. Owing to their adjacent residence, the myCAFs marked by the high expression of α-SMA respond to the increased levels of local TGF-β secreted by cancer cells and promote the production of ECM via the upregulated expression of *α-SMA* and *collagen* genes. In contrast, iCAFs, with much lower α-SMA levels, are distantly located from cancer cells and carry various inflammatory markers, such as IL-1, IL-6, IL-21, C3, CXC-chemokine ligand (CXCL) 1~3, CXCL12, and other inflammatory mediators.^335^ IL-1-mediated JAK/STAT signaling is suggested to regulate the transdifferentiation of CAFs or quiescent PSCs into iCAFs.^335^ With no sign of expressing costimulatory molecules such as CD80 or CD86, apCAFs carrying distinct markers such as major histocompatibility complex (MHC) II, CD74, serum amyloid A3 (SAA3), and secretory leukocyte peptidase inhibitor (SLPI) were inferred to lessen the T-cell response as a decoy receptor activating CD4^+^ T cells.^333,335^ Although it was indicated that myCAFs are profibrotic and immunosuppressive like the other two subtypes in PDAC, studies designed to target the Hh pathway by genetically depleting α-SMA-expressing cells, deleting sonic hedgehog (Shh), or pharmacologically inhibiting Smo did somehow incur some speculation about the tumor-restraining nature of myCAFs, as these manipulations resulted in reduced ECM deposition and α-SMA^+^ cells but surprisingly shortened survival in both preclinical and clinical studies.^333^ However, these observations are skeptical and not entirely indicative of the tumor-restraining roles of myCAFs due to the possibility of other α-SMA-expressing cells accidentally being involved during this pathway-targeted intervention.

Apart from the functional differences among CAF subtypes, emerging evidence from a single-cell RNA sequencing (scRNA-seq) study has illustrated an evolutionary transition of the identity and proportion of distinct CAF subtypes across normal, inflammatory, and premalignant/malignant states.^333^ From the inflammatory to premalignant/malignant state, a vital turning point of carcinogenesis, elevated α-SMA expression, a secretory phenotype, ECM deposition, proliferation, contractility, morphological activation, and deteriorated immunosuppression was observed in fibroblasts.^333^ Likewise, it was demonstrated in *KPC* mice that stromal fibroblasts, defined by positive α-SMA staining, are visible in PanINs and expand during the progression of pancreatic carcinogenesis.^336^ In contrast to normal fibroblasts, which restrain carcinogenesis and cancer progression, CAFs can be adverse via the following mechanisms: 1) increased ECM deposition; 2) enhanced inflammation and angiogenesis; 3) an increased number of tumor-initiating cells (TICs) and secretion of carcinogenic molecules; 4) altered cancer cell signaling, metabolism, and epigenome; 5) the establishment of an immunosuppressive TME; 6) the promotion of cancer cell EMT and stemness; and 10) the supplementation of cancer cells with substantial vital metabolites.^333^

##### CAF-induced immunosuppression

CAFs contribute to carcinogenesis through immunosuppression via their interactions with infiltrating immune cells within the TME. However, further partitioning and analysis of various populations of CAFs indicated the existence of nonnegligible and capricious differences in the function and effect of CAFs during this process.^336^ A recent study also identified CD105 as a robust marker distinguishing two lineages of CAFs with opposite effects on anticancer immunity in PDAC using mass cytometry.^337^ Despite their heterogeneity in biological properties and functions, all subtypes of CAFs can singly participate in immunosuppression. Overall, CAFs not only diminish the recruitment and cytotoxic activity of T cells by secreting immunosuppressive molecules such as TGF-β, IL-1α/β, IL-6, CXCL12, FGFs, EGFs, and TNF-α but also recruit immunosuppressive cells such as MDSCs and TANs that further activate CAFs in turn, and CAFs are also implicated in the recruitment, differentiation, and polarization of TAMs.^336^ In addition to a tumor-restraining phenotype of CAFs, the knockout of *Il17a* favored ECM remodeling and increased the recruitment and activation of CD8^+^ T cells and T_H_1 cells while lowering the numbers of immunosuppressive cells.^338^

Primarily induced by TGF-β, myCAFs impede the infiltration of cytotoxic lymphocytes and reduce the efficacy of anticancer immunity by excessively producing collagens and other components of the ECM, leading to aberrantly enhanced tissue stiffness and increased interstitial fluid pressure (see next section). Furthermore, these physical barriers built up by myCAFs seem to disturb only the activity and functioning of cytotoxic immune cells, such as CD8^+^ T cells, while facilitating the infiltration of immunosuppressive Tregs, MDSCs, and M2-TAMs.^335^ Inflammatory iCAFs, on the other hand, are the primary producers of immunosuppressive cytochemokines and impair anticancer immunity in PDAC.^333^ In addition, inflammatory signals, such as the activation of TLR4, can trigger the transdifferentiation of CAFs into iCAFs and promote the M2 polarization of TAMs and the recruitment of MDSCs, TANs, regulatory B cells (Bregs), and T_H_17 cells while expelling cytotoxic CD8^+^ T cells and hindering NK cells and dendritic cells (DCs), jointly undermining immune surveillance and deteriorating immune evasion.^335^

Beyond the direct regulatory effects of CAFs on immune cells, some other newly found mechanisms are also involved in CAF-induced immunosuppression.^335^ With more direct cell-to-cell contact, the antigens carried by CAFs engage with tumor-infiltrating CD8^+^ T cells and induce programmed cell death due to the subsequent upregulation of programmed cell death ligand (PD-L) 2 and Fas ligand (FasL) on CAFs.^335^ Furthermore, the snatching of nutrients from immune cells by predominantly expanded CAFs might also oppress the normal functioning of immune cells via compromised metabolism, which can be epitomized as the reverse Warburg effect.^335^ In brief, the expansion of CAFs consumes most of the extracellular glucose and produces more lactate, a fuel for anabolic processes for cancer cells, which causes the scarcity of glucose in the TME, damaging the glycolytic activity essential for the competent functioning of T cells in anticancer immunity. Given the immunosuppressive roles of CAFs, the depletion of CAFs expressing fibroblast activation protein (FAP) increased the antitumor efficacy of cytotoxic T cells, diminished the activity of the CAF-secreted cytokine CXCL12, enhanced T-cell recruitment in tumors and acted synergistically with PD-L1 to eradicate cancer cells.^98^ In summary, the gradually increasing and aberrantly expanded CAFs serve as regulators and mediators of the complex crosstalk among various cell types in the TME, most of which, however, promote carcinogenesis.

##### Disrupted ECM deposition

PDAC has more extensive ECM deposition than any other cancer, implying the crucial roles of the composition and function of ECM in initiating PC. Beyond providing structural support for resident cells as a noncellular component within all tissues, the ECM comprises proteins and polysaccharides, maintaining tissue homeostasis through dynamic and reciprocal biochemical and biomechanical interactions among various cellular constituents within the microenvironment.^339^ Structurally, the ECM is divided into the basement membrane (BM) and interstitial matrix (IM). The BM is responsible for supporting the polarity and differentiation of cells, and the IM, in contrast, is the mechanical regulator of tissue homeostasis.^339^ The dynamic physical force and chemical stress within the microenvironment can inevitably have complex effects on the fate of cells, such as inducing pathological conditions related to inflammation, which contributes to pancreatic carcinogenesis by damaging the structure and function of tissues; thus, the dynamic physical force and chemical stress are the major factors of the TME influencing the initiation of cancer.^339^ This process results from the fact that collagen and various ECM components stored in the dense, linearized, and cross-connected tumor-associated stroma (TAS) can stimulate cancer cell proliferation and angiogenesis while suppressing antitumor immunity.^98,339^ Similarly, a previous study also confirmed that ECM components are mainly produced by fibroblasts, and their composition in inflammatory conditions has considerable overlap with that in premalignant and malignant states, accompanied by parallel alterations in accordance with the proportion and function of CAF subtypes.^333^

Unlike normal wound healing, the expansion of CAFs fueled by chronic inflammation results in the accumulation of ECM proteins and the stiffening of the matrix, along with the elevation of cytokines and GFs promoting the proliferation of cancer cells, indicating that force-mediated biochemical signaling can also remodel the surrounding microenvironment and reinforce mechanosignaling.^339^ CAFs are the primary producers of ECM components (e.g., fibrillar collagen, fibronectin, laminin, and proteoglycans such as hyaluronic acid). Fibrosis resulting from chronic and excessive accumulation of ECM components builds a stiff and dense barrier that augments interstitial pressure, collapses blood vessels and reduces the accessibility of oxygen and the infiltration of cytotoxic immune cells while enhancing EMT and supporting tumor growth with biochemical and mechanical fuels in an immunosuppressive TME.^333,336^

The fibrotic and inflammatory microenvironment in obesity and DM leads to momentous mechanical changes in the ECM that promote EMT and tumor growth.^336,340^ Specifically, the TME, rich in collagen and hyaluronic acid, increases the density and stiffness of the PC stroma, shielding cancer cells while reenforcing the inflammatory reaction.^340^ In mice with mutant *Kras*, increased severity of inflammation-induced pancreatic injury and fibrosis were suggested to accelerate carcinogenesis due to the loss of tuft cells and decreased production of prostaglandin D_2_.^341^ Furthermore, obesity is associated with enhanced signaling of angiotensin II type 1 (AT1), which is expressed on both adipocytes and PSCs, with the former also having a high density of AT1 receptor (AT1R) related to the profibrotic pathways and the development of obesity and insulin resistance.^342–344^ As an antifibrotic treatment, the inhibition of AT1 signaling was demonstrated to ameliorate hypoxia and EMT by decreasing the production of IL-1β in adipocytes and PSCs.^246^

##### Hypoxia

Hypoxia is a condition of insufficient O_2_ availability concurrently caused by a reduced vascular density due to the expansion of the desmoplastic stroma and increased demand for O_2_ supply resulting from unbounded oncogene-driven proliferation of cancer cells, which is a common environmental phenomenon in many solid tumors, such as PC. As mentioned above, the extensive desmoplasia and ECM deposition fueled by activation of PSCs causes the compression of blood vessels with relative vessel collapse, decreased blood flow, and consequent hypoxia.^322^ Obesity and T2DM also induce local hypoxia via the overexpression of VEGF, and elevated levels of proangiogenic VEGF-A in patients with obesity and T2DM are correlated with increased vascular density in PDAC and faster disease progression.^98^ In the presence of hyperproliferative cancer cells, the local nutrients and oxygen provided by the vasculature are rapidly exhausted, and the cellular response is consequently activated to withstand hypoxic stress. In contrast to the necrosis of cells distant from the blood supply, substantial changes in transcriptional programs orchestrated by HIF-1 are made as adaptive responses in other cells. As a master transcription regulator, HIF-1 is a heterodimer composed of an α-subunit (HIF-1α) and β-subunit (HIF-1β), with the former being primarily in charge of its activity and stability. Despite its instability and immediate proteasomal degradation under normal levels of oxygen, the stabilization and accumulation of HIF-1α under hypoxic conditions can induce enhanced transcription of numerous genes involved in the modulation of angiogenesis, cell proliferation, survival, inflammation, anaerobic metabolism, cancer initiation and progression.^345^

Consistent with some reports indicating that HIF-1α is not entirely detrimental in all malignancies, pancreas-specific *Hif1a* deletion profoundly accelerated the progression of PanINs with a significant increase in the numbers of intrapancreatic “B1b” B-cell subtypes in *Kras*^*G12D*^-driven murine models,^346^ regardless of the early emergence of HIF-1α during the preinvasive stages of PDAC in both rodents and humans. Nevertheless, the carcinogenic effects of HIF-1α were also observed in PC. In addition to its potential correlations with inflammatory TLRs in PanIN lesions,^347^ HIF-1α can also promote pancreatic carcinogenesis by regulating the expression of EMT-related genes^336^ and its downstream target retention in endoplasmic reticulum sorting receptor 1 (RER1).^348^ Additionally, the transcriptional activity of HIF-1α has also been shown to be correlated with the number of TICs in PC.^349^

In summary, the deviant TME in the context of obesity and DM provides a very favorable growth soil for carcinogenesis. If the final formation of PC is metaphorized into the fruits, then as another decisive factor in carcinogenesis, the alteration of the immune response is undoubtedly the fertilizer for fruit ripening. Next, we will meticulously dissect the contribution of inflammation and the immune response to pancreatic carcinogenesis.

### Inflammation and the immune response

Obesity and DM are characterized by an inflammatory microenvironment, a central and reversible mechanism promoting cancer risk and progression.^251^ Epidemiologically, various inflammatory stimuli or conditions in addition to obesity and DM, such as smoking and chronic pancreatitis, are other risk factors for PC, as both chronic low-grade inflammation and acute or chronic inflammation can be carcinogenic to the pancreas.^350^ All cells in the pancreas are maintained by self-renewal instead of stem cell replenishment owing to the lack of a stem cell population,^351^ with the natural regenerative capacity of the pancreas also endowing a greater chance of slow accumulation of lethal mutations and epigenetic changes in resident cells under inflammatory conditions, eventually leading to carcinogenesis initiated by malignantly dedifferentiated cells.^352^ Despite being protective against tissue damage, it was recently demonstrated that recurrent inflammation in normal pancreatic epithelial cells could cooperate with oncogenic *Kras* to trigger carcinogenesis by inducing irreversible ADM through MAPK signaling, which could be a universal event in the process of pancreatic carcinogenesis.^353^ In addition to ADM,^352^ similar to many other malignancies, the occurrence and progression of PC in the context of obesity and DM implicate insulin resistance, inflammation, and the infiltration of immunosuppressive cells.^354^

Generally, the inflammatory TME of PC comprises stromal cells (mainly fibroblasts and PSCs), endothelial cells, innate immune cells (e.g., macrophages, neutrophils, DCs, and NK cells), adaptive immune cells (T and B lymphocytes), and substantial cytokines and chemokines produced by these cells, jointly triggering tumor initiation and progression in an autocrine and/or paracrine manner. As a member of the inflammatory TME, no component can stay out of the carcinogenic process, although their roles may differ regarding their numbers, distribution, and physiological effects or even playing dual or multiple roles. In the presence of chronic inflammation fueled by the anfractuous interactions among these cells and the secretion of substantial proinflammatory cytokines and GFs, such as ILs, TNF-α, SHh, and TGF-β, along with the activation of the oncogenic networks facilitating the establishment of PanINs and their eventual progression to PC. As a solution, the application of nonsteroid anti-inflammatory drugs has been shown to be protective against PC.^355^

#### Regulation of inflammation-induced pancreatic carcinogenesis

##### Transcription factors

1. *NF‑κB*. NF-κB is a master regulator of innate immunity and inflammation, serving as a molecular bridge between chronic inflammation and cancer development through the modulation of cell proliferation and differentiation as well as the immune response.^356,357^ There are two distinct pathways involved in NF‑κB activation: the canonical and noncanonical pathways. The former is controlled by the IκB kinase (IKK) complex composed of IKKa, IKKb, and IKKc, while the latter is regulated by IKKa and has been reviewed previously regarding its roles in orchestrating inflammation in obesity, DM, and cancer.^358,359^ Despite functioning as a tumor suppressor in precancerous cells by maintaining cellular senescence, NF‑κB acts as a tumor promoter by diminishing immune surveillance during cancer initiation^360,361^ and magnifying RAS activity to fuel the formation of PanINs in the presence of oncogenic *Kras*.^362^ For example, the increased expression of CXCL12 resulting from elevated NF-κB activity in PSCs prevents cytotoxic T cells from infiltrating the tumor and eliminating cancer cells.^363^ Meanwhile, the expression and secretion of many inflammatory cytokines responsive to NF-κB are elevated upon its activation, and the interaction between oncogenic KRAS and NF-κB pathways can promote pancreatic carcinogenesis. Overall, the NF-κB signaling pathway is essential to the development of PDAC induced by mutant *KRAS*.^364^ Once activated, the transcription of growth-promoting genes (e.g., *cyclin D1*, *cyclin E*, *cdk2*, and *c-myc*) and cytokines is increased, and IKKb is also suggested to be necessary for the induction of PanINs and PDAC formation.^364^
  Additionally, other proinflammatory factors regulated by NF-κB, such as IL-1 and TNF-α, also impact NF-κB activity and are strongly associated with most types of PDAC.^365^ IL-1, for instance, induces the constitutive activation of NF-κB, which is capable of promoting cellular transformation,^365,366^ subsequent formation of PanINs,^345,367^ and the determination of the PC phenotype during carcinogenesis.^368^ The regulation of NF-κB in inflammation-related carcinogenesis is at least partially achieved in cooperation with other factors, such as STAT3, and the expression of STAT3 is mediated by NF-κB, which also modulates cytokines and GFs in the TME, controlling the carcinogenic process, cancer cell proliferation and survival.^369^ Notably, various ILs have been reported to be closely associated with STAT3 activation in pancreatic carcinogenesis (discussed later). Similarly, *p21-activated kinase 1* (*Pak1*) acts as an oncogene enhancing tumor formation by binding to fibronectin and interacting with the NF-κB-p65 complex in the *Kras* intact model.^370^ Furthermore, NF‑κB also modulates inflammatory macrophages through the direct regulation of growth and differentiation factor 15 (GDF‑15)/macrophage inhibitory cytokine 1 (MIC‑1), which is highly expressed in PC,^371^ serving as a promoter of early carcinogenesis. Together, these observations support the critical roles of NF-κB in pancreatic carcinogenesis (Fig. 7).

**Fig. 7:**
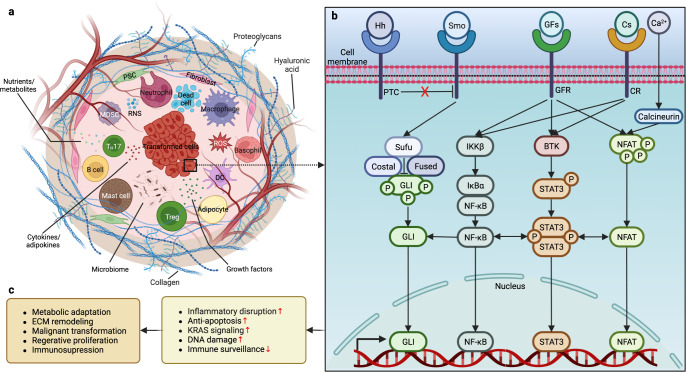
The impacts of the inflammatory microenvironment on pancreatic carcinogenesis. **a** The desmoplastic and inflammatory pancreatic microenvironment in obesity and DM during carcinogenesis comprises transformed cells, stromal cells (mainly CAFs and PSCs), adipocytes, diverse immune cells, dead cells, microbes, metabolites, ROS, RNS, and substantial proinflammatory cytokines/adipokines and GFs, etc., jointly fueling carcinogenesis via constantly activated inflammatory reactions. **b** Similar to other cells in this microenvironment, inflammatory stimuli activate various proinflammatory and oncogenic transcription factors in precancerous cells undergoing malignant transformation. The inhibitory effect of the transmembrane protein PTC on Smo is reversed after its binding to Hh, which increases the release of GLI from their inhibitory complex and favors nuclear translocation. Furthermore, the binding of GFs (e.g., EGF, etc.) and cytokines (IL-1, etc.) to calcineurin can enhance the nuclear translocation and transcriptional activity of NF-κB, STAT3, and NFAT. Bidirectional arrows indicate the interactions among transcription factors. **c** The activation of these transcription factors upregulates the expression of numerous target genes mediating inflammation, cell death, KRAS signaling, DNA damage, and immune surveillance, thereby jointly magnifying the impacts of inflammatory disruption on pancreatic carcinogenesis. BTK Bruton’s tyrosine kinase, Cs cytokines, CR cytokine receptor, DC dendritic cell, ECM extracellular matrix, EGF epidermal growth factor, GFR growth factor receptor, GFs growth factors, GLI glioma-associated oncogene, Hh Hedgehog, IκBα inhibitor of NF-κB subunit α, IKKβ inhibitor of nuclear factor-κB (NF-κB) kinase subunit β, IL-1 interleukin 1, MDSC myeloid-derived suppressor cell, NFAT nuclear factor of activated T cells, NF-κB nuclear factor-κB, P phosphorylation, PSCs pancreatic stellate cells, PTC patched, RNS reactive nitrogen species, ROS reactive oxygen species, Smo Smoothened, STAT3 signal transducer and activator of transcription 3, Sufu suppressor of fused, T_H_ T helper, Treg regulatory T cell. Panel **b** in this figure was adapted from a previous publication^369^
2. *Nuclear factor of activated T cells (NFAT)*. Capable of activating STAT3-like NF-κB, NFAT is another transcription factor family member implicated in pancreatic carcinogenesis.^372^ Likewise, NFAT family members modulate inflammatory processes and the expression of genes controlling cell growth and differentiation.^369^ Therefore, the aberrant activation of NFAT members (NFATc1 and NFATc2) can cause oncogenic transformation through the Ca^2+^/calcineurin/NFAT signaling pathway^369,373^ (Fig. 7).
3. *Glioma-associated oncogene (GLI) family*. GLI family members (GLI1~3) participate in pancreatic carcinogenesis by regulating the expression of genes related to inflammatory reactions, cell proliferation, apoptosis, and autophagy.^369^ As a downstream effector activated by two multitransmembrane proteins, patched (PTC) and Smo, in the Hh signaling pathway, GLI is mainly activated in stromal cells, which indicates that its unique contribution to the TME in pancreatic carcinogenesis is paracrine signaling^369^ (Fig. 7). However, this contribution is not entirely Hh-dependent.^374^ Moreover, KRAS was shown to induce pancreatic carcinogenesis by increasing GLI1 expression and protein stability while cooperating with GLI2.^369^ Collectively, the increased expression of both GLI1 and GLI2 also impacts PanIN lesions through an SMO-independent mechanism regulated by TGF-β.^374,375^

Other less-studied transcription factors also play different roles in pancreatic carcinogenesis. For example, the homeodomain transcription factor Prox1, which regulates mouse exocrine pancreas development, was illustrated to be transiently reactivated in acinar cells undergoing dedifferentiation and ADM. However, the expression of *Prox1* was absent in the neoplastic lesions and tumors in the pancreas of both mice and humans. Furthermore, Prox1 heterozygosity drastically increased the formation of ADM and early neoplasia, with concurrent enhancement of inflammation in mice carrying oncogenic *Kras*.^376^

#### Cytokines

A variety of cytokines are secreted into the TME in response to inflammation and the immune response, modulating tumor development and progression. While many cytokines are secreted to inhibit carcinogenesis by the host, cancer cells can use these “double agents” to promote cell growth, reduce apoptosis, and facilitate tumor formation.^377^

##### Interleukins (ILs)

Chronic inflammation can lead to the overproduction of proinflammatory cytokines, and ILs are essential molecules downstream of NF‑κB signaling, playing crucial roles in promoting pancreatic carcinogenesis. Given their diversity and complexity in physiological functions, ILs can drive pancreatic carcinogenesis by enhancing inflammatory signaling, angiogenesis, and immunosuppression; dysregulating cell proliferation, survival and death; disrupting autophagy; and promoting ER stress, EMT and cell transformation (Table 1). To date, studies connecting ILs to pancreatic carcinogenesis are still lacking in comparison with their large numbers and considerable varieties. Given the diversity of ILs in cellular sources, receptors, signaling pathways and physiological functions, their roles in pancreatic carcinogenesis need to be further explored to fully illustrate the carcinogenic effects of these critical regulators of anticancer immunity.

**Table 1:** ILs related to pancreatic carcinogenesis

Interleukins	Source (not exhaustive)	Receptor	Inflammatory impact	Effect on immune response	Mechanism in pancreatic carcinogenesis	Refs
***IL-1 superfamily: IL-1 subfamily***						
IL-1α	Keratinocytes, epithelial cells, cells from the entire gastrointestinal tract, brain astrocytes, myeloid cells	IL-1R1 IL-1R3 sIL-1R3	↕	General function ➀ Extensive regulation of immune response, differentiation, and activation of myelomonocytic cells, function of Tregs ➁ Maintenance of homeostasis and pathogenesis of diseases and cancer Cancer immunity ➀ T_H_17 polarization ➁ Recruitment of MDSCs, TAMs and TANs ➂ T-cell activation via the production of IL-2 and IL-2R	Activation of NF-κB signaling and CAFs to maintain inflammatory TME	^28,246,364,394,491–505^
IL-1β	Myeloid cells	IL-1R2 IL-1R3 sIL-1R2 sIL-1R3	↓		➀ Increased infiltration of immunosuppressive macrophages and MDSCs ➁ Enhanced angiogenesis ➂ Prolonged cell survival ➃ Activation of quiescent PSCs and suppression of antitumor immunity via TLR4 signaling and NLRP3 inflammasome ➄ Dysregulated autophagy and increased ER stress	
IL-33	Endothelial, epithelial, and fibroblast-like cells	IL-1R3-IL-1R4 sIL-1R4	↓	➀ Stimulation of myeloid and lymphoid cells supporting their proliferation, survival, migration, and production of type 2 immune mediators (IL-5 and IL-13) ➁ Direction of DC-mediated T_H_2 cell polarization ➂ Supporting type 1 responses dominated by TNF and IFNγ	Driver of early-stage neoplasia via chromatin switching following tissue damage	^495,506,507^
***IL-2 (common γ-chain) family***						
IL-4	Epithelial cells, CD4^+^ T cells, basophils, eosinophils, mast cells, macrophages, DCs, NK cells, NKT cells	IL-4Rα-γc IL-4Rα-IL-13Rα1	↓	➀ Differentiation of naïve CD4 T cells into T_H_2 cells ➁ Ig class switch to IgG1 and IgE in B cells ➂ Alternative macrophage activation	➀ Activation of c-Jun, ERK-1/2, and STAT3 signaling ➁ Promoted cell growth and survival via decreased apoptosis	^495,508–515^
***IL-3 family***						
IL-5	T cells (mainly T_H_2 cells), eosinophils, mast cells, ILCs	IL-5Rα-βc	↑	➀ Chemotaxis and activation of the integrin CD11b and prolonging eosinophil survival ➁ Antibody secretion and class switching of B cells	Enhanced pancreatic tumor motility through STAT5 activation	^495,516–518^
***IL-6 family***						
IL-6	Immune and nonimmune cells	Classic: IL-6Rα-gp130 Trans: sIL-6Rα-gp130	↓	Regulation of innate and adaptive immunity via B-cell stimulation and regulation of the balance between Tregs and effector T cells	➀ Activation of STAT1, STAT3, MAPK, and PI3K signaling and other oncogenic pathways ➁ Increased cell proliferation and decreased apoptosis ➂ Dysregulated autophagy	^28,385,446,495,514,519–527^
***IL-10 family***						
IL-22	CD4^+^ helper T cells, CD8^+^ cytotoxic T cells, γδ T cells, ILCs	IL-22Rα1-IL-10Rβ IL-22Rα2 (IL-22BP)	↓	Regulation of innate and adaptive immunity and tissue homeostasis both locally and systemically	➀ Activation of STAT1, STAT3, STAT5 and NF-κB, MAPK, and PI3K/Akt/mTOR signaling pathways ➁ Promoted cell proliferation and survival, ADM, and the EMT and stemness of precancerous cells	^495,528–535^
***IL-12 family***						
IL-35	CD4^+^Foxp3^+^ Tregs, Bregs, DCs, active endothelial and muscle cells, monocytes	IL-12Rβ2-gp130 IL-12Rβ2-IL-12Rβ2 gp130-gp130 IL-27Rα-IL-12Rβ2	↓	➀ T-cell proliferation and expansion↓ ➁ T_H_17 cell differentiation↓ ➂ Number of T_H_ cells in humoral immunity↓ ➃ Expansion and activation of Tregs↑ ➄ Expression of IFNs↑ ➅ Transformation of B cells into IL‐35‐producing Bregs	Stimulated cell proliferation and diminished immune response through the production of IL-10, IL-35, and activation of BTK signaling	^411,495,523,536^
***IL-17 family***						
IL-17A/F	T_H_17 cells, CD8^+^ T cells, γδ T cells, NKT cells, ILCs	IL-17RA–IL-17RC	↑	Host defense against bacterial and fungal pathogens	➀ Promotion of neoplastic cell transformation, PanIN formation and progression ➁ Activation of CAFs ➂ Cancer cell stemness↑ ➃ Increased cell growth and decreased cell death through activation of the gp130-JAK2-STAT3-dependent pathway during ADM	^28,335,338,495,537–543^
IL-17B	Intestinal and pancreatic cells	IL-17RB	↕	➀ Enhanced inflammatory response via neutrophil migration ➁ Anti-inflammatory effect via blocked IL-25 signaling		
IL-17C	Epithelial cells	IL-17RA-IL-17RE	↑	Protection against microbial infection and pathogenesis of autoimmune disease		
IL-17D	Resting CD4^+^ T cells, CD19^+^ B cells, T cells, smooth muscle cells, epithelial cells	Unknown	↑	➀ Classic proinflammatory-cytokine responses ➁ Tumor and viral surveillance mediated by NK cells		
IL-17E (IL-25)	CD4^+^ cells, CD8^+^ T cells, macrophages, DCs, mast cells, eosinophils, epithelial cells	IL-17RA-IL-17RB	↓	Type 2 immunity and host defense against helminth and parasitic infections via production of anti-inflammatory cytokines such as IL-4, IL-5, and IL-13 for eosinophil recruitment		
***Other ILs***						
IL-8 (CXCL8)	Endothelial cells, fibroblasts, keratinocytes, synovial cells, chondrocytes, infiltrating neutrophils, TAMs, T cells	CXCR1 CXCR2 ACKR1/DARC	↑	Recruitment of neutrophils and MDSCs	Promotion of cell growth, survival, and angiogenesis	^495,544–549^
IL-13	CD4^+^ T cells, basophils, eosinophils, mast cells, macrophages, DCs, NK cells, NKT cells	IL-13Rα1-IL-4Rα IL-13Rα2	↓	➀ Suppressing the production of proinflammatory cytokines ➁ Enhancing major histocompatibility complex class II and CD23 on monocytes ➂ Induction of anti-CD40-dependent IgG class switching ➃ Promoting IgG and IgM synthesis in B cells	➀ Detrimental polarization of TAMs in ADM/PanIN lesions ➁ Exacerbated pancreatic fibrosis via the release of IL-1Rα and CCL2	^27,495,513,515^

*ADM* acinar-to-ductal metaplasia, *Akt* protein kinase B, *Bregs* regulatory B cells, *BTK* Bruton’s tyrosine kinase, *CAFs* cancer-associated fibroblasts, *CCL* CC chemokine ligand, *CXCL* CXC-chemokine ligand, *CXCR* CXC-chemokine receptor, *DCs* dendritic cells, *EMT* epithelial-mesenchymal transition, *ER* endoplasmic reticulum, *ERK* extracellular signal-regulated kinase, *IFNs* interferons, *Ig* immunoglobin, *ILCs* innate lymphoid cells, *MAPK* mitogen-activated protein kinase, *MDSCs* myeloid-derived suppressor cells, *mTOR* mammalian target of rapamycin, *NK* natural killer, *NKT* natural killer T, *NLRP3* NLR family pyrin domain containing 3, *PanIN* pancreatic intraepithelial neoplasia, *PI3K* phosphatidylinositol-3-kinase, *PSCs* pancreatic stellate cells, *STAT* signal transducer and activator of transcription, *TAMs* tumor-associated macrophages, *TANs* tumor-associated neutrophils, *T*_*H*_ T helper, *TLR4* Toll-like receptor 4, *TME* tumor microenvironment, *TNF* tumor necrosis factor, *Tregs* regulatory T cells

##### Growth factors (GFs)

Accumulating evidence has suggested that various types of GFs are involved in pancreatic carcinogenesis, including TGFs, EGFs, HGFs, IGFs, VEGFs, PDGFs and FGFs.^378^ Functionally, GFs are secreted as signaling polypeptides capable of regulating specific cellular responses (e.g., cell proliferation, survival, migration, and differentiation), metabolism, and other cell functions by binding with specific and highly compatible cell membrane receptors. Several types of GFs produced by immune cells or other cells in the TME play crucial roles in inflammation-related carcinogenesis.

##### Transforming growth factors (TGFs).

TGFs are members of the EGF family that act synergistically to induce anchorage-independent growth of target cells. Two major types of TGFs, TGF-α and TGF-β, are known to be correlated with pancreatic carcinogenesis. A previous study confirmed that TGF-α is involved in ADM during the early stage of pancreatic carcinogenesis and local progression of PC,^379–382^ and it was demonstrated in 3D explant culture of primary pancreatic acinar cells that protein kinase D1 (PKD1), a downstream target of TGF-α and KRAS, is a mediator of ADM by activating the Notch signaling pathway.^383^ The combination of *smad4* loss and *tgfα* overexpression was also shown to promote inflammation, ADM, fibrosis, and the formation and progression of PanIN lesions.^384^

TGF-β, on the other hand, acts as a tumor suppressor at the early stage of pancreatic carcinogenesis but promotes cancer progression later on.^385^ In detail, the activation of TGF-β forms the SMAD2/3/4 complex, which regulates the cell cycle, apoptosis, and differentiation to prevent carcinogenesis. However, the additional mutation and loss of *SMAD4* leads to the functional switch of TGF‑β and drives carcinogenesis.^385^

##### Vascular endothelial growth factor (VEGF).

Apart from targeting endothelial cells, VEGF has been shown to affect multiple cell types.^386^ As its name implies, VEGF is vital in angiogenesis and is also involved in tumor formation and growth through the modulation of vascular permeability, which is regulated by HIF and other hypoxia-related genes as well as other oncogenic mutations.^386^ In addition, partly dependent on MEK1/2 and JNK signaling, the activation of KRAS was suggested to initiate angiogenesis through paracrine epithelial secretion of CXC chemokines and VEGF,^387^ and MMP-9 is one of the regulators of this angiogenic switch during carcinogenesis.^388^ VEGF also participates in EMT, and it was suggested that VEGF has clinical potential for predicting malignant transformation in patients with IPMN,^389^ with its expression also being correlated with metastasis and poor prognosis.^390^

##### Fibroblast growth factors (FGFs)

FGFs were given their names because they promote the proliferation of fibroblasts, and more than 20 members of this family have been discovered thus far. FGFs modulate several downstream pathways, such as the PI3K/AKT/mTOR and MAPK pathways, to regulate cell proliferation, differentiation, survival, migration, invasion, metastasis, angiogenesis, and wound repair, thereby being implicated in pancreatic carcinogenesis.^378^ However, not all FGFs are carcinogenic, and FGF21, a metabolic regulator preventing obesity, was shown to be suppressed in mice fed an HFD and carrying oncogenic *kras* mutations, which led to extensive inflammation, pancreatic cysts, PanIN, and eventual PDAC.^42^

##### Other chemocytokines and GFs.

TNF-α, a type II transmembrane protein with signaling potential released by proteolytic cleavage, is another central regulator of inflammation with dual roles in the TME related to carcinogenesis.^28^ The two binding proteins of TNF‑α, TNFR1 and TNFR2, have distinctly opposite roles in inflammatory reactions. Ubiquitously expressed TNFR1 (also known as p55) activates NF‑κB, JNK, and p38‑MAPK as a promoter of inflammation. In contrast, TNFR2 (also known as p75) is mainly expressed in immune cell receptors and regulates anti‑inflammatory signaling.^28,385^ The pro- or antitumoral effect of TNF‑α, of note, also depends on its local concentration and expression site, as it was demonstrated that TNF‑α can be carcinogenic by inducing DNA damage via enhanced production of ROS and reactive nitrogen species (RNS), especially at a low concentration.^28^ More importantly, TNF‑α can also be produced by cancer cells, which further stimulates the secretion of other cytokines and chemokines in the TME to exacerbate inflammation and immunosuppression, promoting tumor growth, angiogenesis, and metastasis.^385^

In contrast to the abovementioned GFs with protumor effects, PEDF has a tumor-suppressing effect on PC and limits pancreatic carcinogenesis by decreasing intratumoral inflammation and fibrosis.^391^ In addition, other components of inflammation also participate in carcinogenesis. Necroptosis (programmed necrosis) plays a vital role in the development of PC. It was shown that the major components of the necrosome, receptor-interacting protein (RIP) 1 and RIP3, were associated with oncogenic progression and immunosuppression mediated by the chemokine attractant CXCL1 and cytoplasmic SAP130 (a subunit of the histone deacetylase complex).^392^

Having a functional similarity with CXCL1, CXCL12 is one of the most studied chemokines in pancreatic carcinogenesis; CXCL12 is the ligand of CXCR4 receptors and is capable of enhancing tumor growth while inhibiting immune surveillance through local autocrine and paracrine mechanisms.^385^ Likewise, it was demonstrated both in vivo and in vitro that NF-κB signaling in PSCs increases the expression of CXCL12, which promotes tumor growth by diminishing the infiltration of cytotoxic T cells and the eradication of cancer cells.^363^ In addition, CXCL12 also promotes EMT, with the activation of CXCR4 increasing the expression of Smo, GLI1, and EMT markers to establish a fibrotic and hypovascular microenvironment in PC.^385^

#### Proteins involved in inflammation and the immune response

##### Toll-like receptors (TLRs)

TLRs are type I membrane receptors and the PRRs of the innate immune system; they are involved in the pathogenesis of PC by maintaining an inflammatory microenvironment and mediating the interactions between environmental stimuli and innate immunity.^385,393^ TLR signaling has been proven to promote carcinogenesis and cancer progression. For example, the overexpression of TLR2, TLR4, and TLR9 was detected in PanIN and PDAC lesions in resected human samples, with a linear increase in the expression of TLR2 from PanIN1 to PanIN3, TLR4 expression being the highest in inflamed ducts, and TLR9 expression in PanIN1 lesions.^347^ In a preclinical study, the endogenous microbiome of PDAC was shown to create an immunosuppressive environment by inhibiting T cells through selective TLR ligation.^207^

Overall, multiple members of the TLR family participate in pancreatic carcinogenesis. Driven by microbial-dependent activation of TLR4 signaling and subsequent engagement of the NLRP3 inflammasome, IL-1β secreted by PDAC cells supports the establishment of the carcinogenic TME by promoting the activation and secretory phenotype of quiescent PSCs and suppressing the immune response mediated by M2 macrophages, MDSCs, CD1d^hi^CD5^+^ Bregs, and T_H_17 cells.^394^ TLR2 and other TLR family members also exert a protumorigenic effect on the pancreas.^395^ In the context of enhanced inflammation through the activation of STAT3, Notch, NF-κB, and MAPK signaling, along with elevated expression in both the epithelial and stromal compartments of PC in humans and rodents, TLR7 promotes tumor progression by leading to the loss of *phosphatase and tensin homolog (PTEN)*, *CDKN2A*, and *cyclin D1* and the upregulation of *CDKN1A*, *CDKN1B*, *TP53*, *c-Myc*, SHPTP1, TGF-β, PPAR-γ, and cyclin B1.^393^ Similarly, the activation of TLR9 augments proinflammatory signaling in transformed epithelial cells, and its ligation promotes epithelial cell proliferation and fibrosis through the regulation of CCL11 on PSCs.^396^ Furthermore, TLR9 can be immunosuppressive by fueling the recruitment of Tregs and the proliferation of MDSCs.^396^ Apart from TLRs, NLR1 and NLR2, as bacterial sensors, function against gut inflammation and carcinogenesis in obesity.^397^

##### Cyclooxygenase 2 (COX2)

COX2 is a key enzyme that responds to multiple cytokines and GFs implicated in inflammation. Various binding elements have been found within the COX2 promoter for *TP53*, *NF‑κB*, and other transcription factors.^385^ Regarding its roles in pancreatic carcinogenesis, it was shown in mice harboring *Kras* mutations that the activation of COX2 accelerates the progression of PanIN via NOTCH1 signaling.^398^ Diet-induced obesity in mice can activate oncogenic *Kras* via COX2, leading to pancreatic inflammation, fibrosis, and the development of PanINs to PDAC.^40^ Additionally, COX2 was suggested to be indispensable for the positive feedback loop regulated by NF-κB, which prolongs Ras signaling and fuels chronic inflammation and PanIN formation in mice expressing physiological levels of oncogenic *Kras*.^362^ The coactivation of COX2 and KRAS was shown to accelerate the progression of PanIN lesions,^398^ while the inhibition of COX2 was demonstrated to abolish chemically induced pancreatic carcinogenesis in hamsters.^399^ Moreover, COX2 can be carcinogenic by suppressing the cell-competition-mediated apical elimination of Ras^V12^-transformed cells, which are the initiators of pancreatic carcinogenesis.^400^

##### Bromodomain and extra terminal domain (BET) proteins

BET proteins are members of a bromodomain subfamily including BRD2~4 and BRDT, which mediate histone acetylation recognition, chromatin remodeling, and transcription regulation, therefore regulating inflammation, apoptosis, cell proliferation, the cell cycle, and cancer.^98^ In addition to enhancing the transcription of NF-κB-dependent proinflammatory cytokine genes, BET proteins also regulate the STAT signaling pathway.^98^

#### Immune cells

Disrupted metabolism and constantly activated inflammatory pathways provide preferable support for cancer initiation. During this process, substantial metabolic, endocrine, and inflammatory mediators as well as the intricate interactions among cells fuel malignant transformation, while the original metabolic and functional characteristics of immune cells are also reshaped. Metabolic disorders stimulate the expansion of immunosuppressive and protumorigenic immune cells, and the accumulation of these cells establishes a TME perfectly suitable for tumor formation.^401^

##### Leukocytes

Obesity and DM are associated with various immunological changes, such as leukocytosis resulting from chronic inflammation in adipose tissue,^402^ and the infiltration of different leukocytes has distinct effects on pancreatic carcinogenesis and progression.^200^

1. *Lymphocytes*. The impact of obesity and DM on the immune system is quite complicated. This impact not only alters the production of proinflammatory cytokines in the TME but also involves many other parts of the immune response, such as the adaptive immune response. Various cell types, including MDSCs, macrophages, T_H_17 cells and other CD8^+^ T cells, are present in the TME of *Kras*-mutant PDAC mice during the early stage of pancreatic carcinogenesis.^98^ In particular, peripancreatic leukocytes can either be protective against carcinogenesis by restraining tumor growth via antigen-restricted tumoricidal immune responses or, conversely, promote tumor progression through the induction of immunosuppression.^403^
  - *T cells*. Both obesity and DM have been shown to induce negative impacts on the population and function of T cells.^404^ As a vital part of the adaptive immune response, T cells are the pioneers of anticancer immunity and are supposed to be on the front lines preventing carcinogenesis. However, the incompetence of immune surveillance and evasion of precancerous cells indicate that there might be suspicious “traitors” among this defensive line. Indeed, the infiltration of distinct subpopulations of T cells induces different impacts on the carcinogenic process depending on their types, spatial distribution, and accompanying macrophage infiltration.^335^ In detail, as the predominant population of T cells in humans and a group of non-MHC-restricted lymphocyte subsets closely related to innate immunity, γδT cells, accounting for approximately 40% of the tumor-infiltrating T cells in human PDAC, have been shown to promote pancreatic carcinogenesis by mitigating the immune infiltration, activation, and T_H_1 polarization of αβT cells.^403^ In inflammation-driven pancreatic carcinogenesis, it was revealed that CD4^+^ T cells promote the establishment of an immunosuppressive TME and favor PanIN formation by mitigating the antitumor effects of CD8^+^ T cells, including the production of the cytotoxic molecules IFN-γ, TNF, perforin, and granzymes.^335,405^
    The subpopulations of CD4^+^ T cells are made up of immune-activating and IFN-producing T_H_1 (T-BET^+^) cells and immunosuppressive T_H_2 (GATA3^+^) cells.^335^ While the former enhances immunity against intracellular pathogens and tumors by producing IFN-γ and other cytotoxic molecules to activate and recruit cytotoxic T cells, M1 macrophages, and NK cells, the latter secretes anti-inflammatory cytokines, such as IL-4, IL-5, and IL-13, and diminishes the antitumor response by inducing the M2 phenotype of macrophages and increasing the proliferation of PC cells via enhanced activation of TLRs and STAT3/AKT/MAPK signaling, which is jointly mediated by cancer cells, CAFs, TAMs, DCs, and B cells.^335^ Hence, in contrast to the inhibition of pancreatic tumor growth fueled by T_H_1-polarized CD4^+^ and CD8^+^ T cells, antigen-specific T_H_2-polarized CD4^+^ T cells were shown to promote PC progression in rodents.^200^
    The main subpopulation of CD8^+^ T cells is cytotoxic T cells producing IFN-γ, TNF, and other cytotoxic molecules and playing critical roles in antitumor immunity. It was suggested that the functioning of cytotoxic T cells could be diminished by the increased expression of CXCL12 via the enhanced activity of NF-κB in PSCs.^363^ In addition, cancer cells can also sabotage the function of CD8^+^ T cells by reducing their survival and increasing programmed death 1 (PD-1), PD-L1, and cytotoxic T lymphocyte-associated protein 4 (CTLA-4).^335^ In contrast, the depletion of myeloid cells prevents mutant *Kras*^*G12D*^-driven pancreatic carcinogenesis, which is induced by enhancement of the antitumor activity of CD8^+^ T cells and elevated expression of PD-L1 in tumor cells in an EGFR/MAPK-dependent manner.^406^ Moreover, the oncogenic *Kras*^*G12D*^-dependent upregulation of granulocyte-macrophage colony-stimulating factor (GM-CSF) present in both the pancreatic ductal epithelial cells (PDECs) of mice and human PanIN lesions is essential for the recruitment of Gr1^+^CD11b^+^ myeloid cells and the establishment of an immunosuppressive TME, which causes PC cells to evade CD8^+^ T-cell-driven antitumor immunity.^407^
    Through their production of multiple cytokines, including IL-17, IL-21, and IL-22, depending on the lineage-specific transcription factor RORγt, T_H_17 cells and Tregs are also involved in pancreatic carcinogenesis. Fueled by oncogenic KRAS, MAPK signaling induces the accumulation of T_H_17 cells in the TME and the subsequent upregulation of IL-17R to drive the progression of PanIN to PDAC in a cell-autonomous manner via REG3G, a member of the regenerating islet-derived gene (REG) 3 protein family that is associated with innate immunity.^335^ Moreover, REG3G promotes tumor growth, the differentiation of Tregs and the recruitment of MDSCs while inhibiting the maturation of DCs and hindering the activity of CD8^+^ T cells by interacting with PD-1/PD-L1 through the JAK2/STAT3 signaling pathway in DCs.^408^ Similarly, the ablation of the IFN-stimulated gene 15 (ISG15) pathway, which is highly active in PDAC, was demonstrated to suppress the expression of PD-L1 and the activity of Tregs while increasing the number of CD8^+^ tumor-infiltrating T cells, thereby mitigating pancreatic carcinogenesis.^409^ Similarly, the linear increase in the numbers of Tregs from precursor lesions to the progressed PC raised the speculation that Tregs are responsible for immunosuppression and a culprit for pancreatic carcinogenesis as a critical source of TGF-β ligands in the TME.^335^ However, the depletion of Tregs accelerated tumor progression by reshaping fibroblasts and increasing CCL3, CCL6, and CCL8, which augmented myeloid cell recruitment and immune suppression and promoted carcinogenesis.^410^ These contradictory results indicate that whether Tregs are promotive or inhibitive in pancreatic carcinogenesis may depend on a variety of factors. Based on the existing results, it would be hasty to assert the roles of Tregs, which need to be revealed by future studies.
  - *B cells*. As another important part of adaptive immunity, the distribution of B cells within the TME is often deemed to induce protumorigenic and immunosuppressive effects. In contrast to the perspective that HIF-1α is the main contributor to the hypoxic TME that promotes pancreatic carcinogenesis, the pancreas-specific deletion of *Hif1α* profoundly accelerated the development of PanINs in mice carrying mutant *Kras*^*G12D*^ with a concurrent accumulation of intrapancreatic B lymphocytes, featured by prominent influx of a rare “B1β” B-cell subtype.^346^ Notably, a unique subtype of B cells, CD1d^hi^CD5^+^ Bregs, accumulate in the pancreatic parenchyma during early neoplasia and accelerate the development of PC in mice by stimulating the proliferation of cancer cells, which is mediated by the expression of IL-35.^411^ Later, another study showed that the inhibition of Bruton’s tyrosine kinase (BTK), a critical B-cell kinase, disrupts the differentiation of CD1d^hi^CD5^+^ Bregs and the production of IL-10 and IL-35 in the pancreatic TME, followed by an increase in stromal CD8^+^IFNγ^+^ cytotoxic T cells and the cessation of PanIN progression.^412^
  - *NK cells*. Representing 5~20% of circulating lymphocytes in humans, NK cells have long been known to participate in the antitumor immune response and reduce carcinogenesis.^413^ NK cells not only act as a component of anticancer immunity by nonspecifically recognizing and directly killing tumor cells but also regulate and shape the antitumor immune landscape through crosstalk with other cells, including DCs, macrophages, T cells, and endothelial cells.^335^ Emerging evidence has indicated that some signaling molecules within the TME can favor the induction of immune evasion from NK cells while inducing a tumor-promoting phenotype.^335^ However, unlike the decreased CD8^+^ T cells, the circulating levels of NK cells (CD56^+^CD3^-^) are elevated in patients with PDAC, which is positively correlated with a longer survival, although the cytotoxicity of NK cells in PDAC patients is weaker than that in healthy individuals owing to the reduction in cytotoxic mediators and increase in immunosuppressive signaling molecules, such as TGF-β, IL-10, IL-18, indoleamine 2,3-dioxygenase (IDO), and MMPs.^335,414^
    Overall, apart from impaired cell recognition, the recruitment and infiltration of NK cells within the TME are reduced in PC.^414^ The activated PSCs in the desmoplastic stroma of PC exile NK cells from the local TME, partially accounting for the extremely low frequency (<0.5%) of NK cell infiltration in PDAC tumors, while NK cells isolated from tumor samples exert their intrinsic anticancer effects.^335^ Similar exciting findings inspired researchers to come up with the concept called “NK cell restoration”, aiming to reactivate the suppressed cytotoxic NK cells within the TME as a strategy of immune therapy.^335^ However, the extremely low numbers of NK cells in the TME of PC make it harder to depict the roles of NK cells during the early stage. Therefore, the gap in knowledge in this field awaits future efforts to reveal the roles that NK cells play and the changes they undergo during the carcinogenesis and progression of PC.
  - *Dendritic cells*. DCs (or S100^+^ cells) are clusters of specialized APCs capable of inducing antigen-specific immune responses and participating in antitumor immunity, especially myeloid DCs (mDCs, also known as CD11b^+^ cells).^415^ Quantitatively, DCs are rare in the TME of PDAC but are mainly located in the stroma adjacent to the tumor. It was suggested that a higher number of DCs, whether in the circulation or within the tumor, is correlated with longer survival, regardless of the stage of PC at diagnosis.^415^ Of note, the number of DCs infiltrated within the tumor is parallel to the number of CD4^+^ and CD8^+^ T cells in patients with PDAC, implying the presence of all key components in the antitumor immune response, and the decreased number and function of DCs in PDAC is responsible for the immune tolerance resulting from compromised antigen presentation.^415^

Other studies also confirmed the participation of DCs in pancreatic carcinogenesis. For example, the systemic and progressive dysfunction of type 1 conventional DCs (cDC1s) was found to occur in the earliest stages of preinvasive PanIN in *KPC* mice, which was fueled by increased apoptosis and production of IL-6 in the TME that subsequently leads to the disruption of cDC1 maturation.^416^ During pancreatic carcinogenesis in IPMN and intraepithelial precursor lesions that progress from adenoma to carcinoma, the switch from an antitumor immune response to immune tolerance occurs between the stages of intraductal papillary mucinous adenoma (IPMA) and intraductal papillary mucinous carcinoma (IPMC), where the increased expression levels of CXCL17 and intercellular adhesion molecule 2 (ICAM2) first enhance the infiltration and accumulation of immature mDCs while promoting the susceptibility of the tumor cells to cytotoxic T-cell-mediated cytolysis, which then disappears in IPMC.^417^

##### Granulocytes

1. *Neutrophils*. As the most abundant circulating leukocytes in humans, neutrophils are increased in WAT and produce the serine protease neutrophil elastase that cleaves insulin receptor substrate 1 (IRS1), preventing IRS1 from binding to PI3K and leading to insulin resistance,^418^ and the activation of the PI3K pathway is also important in pancreatic carcinogenesis in obesity and DM. Beyond that, accounting for an essential part of the innate immune system, the enormous population of neutrophils is inevitably implicated in the pathogenesis of various diseases, including the development and progression of cancer, such as PC.^419–421^ Characterized by their versatile phenotypes and functionality, TANs infiltrating tumors play crucial and decisive roles in carcinogenesis, and clinical research has shown a correlation between the elevation of the neutrophil-lymphocyte ratio and the stage and/or aggressiveness of cancer.^420^ TANs can be classified into two types based on polarization states, antitumor N1 neutrophils and protumor N2 neutrophils, and the transformation of both types of TANs with unique markers can be induced by multiple kinds of signaling cytokines and chemokines.^421^ It was also confirmed that the transformation of TANs from N1 to N2 can promote carcinogenesis in a hypoxic and immunosuppressive TME.^420,422^
  Additionally, emerging evidence has shown that neutrophils have a longer life cycle and are capable of producing various cytokines and chemokines, affecting the TME by acting on both cancer cells and stromal cells.^420^ Some of these biologically active molecules can be mutagens or be in other forms to promote carcinogenesis through augmented cell proliferation, survival, migration, and angiogenesis in various cancers.^420^ As mentioned above, the complex and changeable phenotypes and functionality of neutrophils also have a two-sided influence on antitumor immunity. On the one hand, despite not being able to kill cancer cells solely or directly, neutrophils show an antitumor effect when stimulated by various cytokines and chemokines or kill cancer cells with indirect cytotoxicity, while they also interact with other immune cells, such as T cells and DCs, to inhibit carcinogenesis by enhancing the immune response.^420^ On the other hand, neutrophils also contribute to immunosuppression by recruiting immunosuppressive cells, such as Tregs and MDSCs, and diminishing the activity of T cells and NK cells via the formation of neutrophil extracellular traps (NETs).^420^
  Mechanistically, neutrophils can be recruited to the TME by a substantial number cytokines and chemokines via various signaling pathways, and the abolition of these pathways has shown a significant antitumor effect in different animal models.^421^ Moreover, neutrophils can interact with other components of the TME, such as T cells, fibroblasts, PSCs, and macrophages, to participate in carcinogenesis and the subsequent development of PC by influencing the immune status.^421^ Finally, the formation of NETs has been implicated in the progression and metastasis of different types of cancer, yet its roles in pancreatic carcinogenesis have rarely been reported. Thus, research in this field will undoubtedly bring many interesting discoveries in the future.
2. *Basophils*. The number of basophils is far less than that of neutrophils, accounting for only <1% of human peripheral leukocytes. Like neutrophils, basophils also develop from hematopoietic stem cells in bone marrow via the stimulation of IL-3, the most important growth factor for the development of basophils in both humans and mice.^423^ Except for this common phenotypic marker IL-3, basophils of humans and mice express a variety of unique markers of their own, which basically modulate the inflammatory and immune response through the secretion of cytokines and chemokines, promoting or mitigating carcinogenesis.^423^ Accumulating evidence has suggested that not only do basophils reside in tissues rather than circulating in peripheral blood, but tissue-resident basophils also possess a specific phenotype shaped by the tissue microenvironment that is vital for the roles they play and the impacts they have on the development of cancer.^423^
  Basophils are also present in the TME of PC, and beyond producing a variety of angiogenic factors, the receptors expressed on the surface of basophils also allow them to be regulated by some of these factors and participate in angiogenesis during carcinogenesis.^423^ Using an orthotopic model of PC, it was shown that basophils are indispensable for the formation of PC, and the interaction between basophils and Tregs contributes to immune tolerance in the TME of PC via the mediation of several proinflammatory cytokines, such as IL-3, IL-4, IL-8, and IL-13.^423^
3. *Macrophages*. Macrophages are the most abundant cell type in the TME, and they affect tumor biology in different ways. As macrophages are highly plastic, the macrophages in the ATME can be phenotypically, spatially, and functionally heterogeneous, playing a vital role in maintaining tissue homeostasis and mediating immunometabolism. Although disputable, it is clear that macrophages are a group of multisource cells originating from hematopoietic stem cells, yolk sac cells, adult monocytes, and fetal monocytes.^424^ Originating from embryonic development as the main source of pancreas-resident macrophages, a vast number of TAMs are recruited during PDAC progression, when they expand and exhibit a profibrotic profile fueling cancer-related inflammation, immune evasion, and matrix remodeling.^336^
  A previous study confirmed that the infiltration and activation of macrophages are required for pancreatic carcinogenesis.^425^ The coculture of PDECs and macrophages supplemented with high-level glucose showed a significantly promoted malignant transformation of PDECs via enhanced EMT, suggesting a supporting role of macrophages in DM-related pancreatic carcinogenesis.^426^ Similar to TANs, TAMs are capable of making adaptations by switching their specific phenotype and differentiating into the M1 or M2 phenotype in response to various microenvironmental stimuli, with the latter commonly being deemed tumorigenic by contributing to the establishment of a protumor TME and promoting inflammation, angiogenesis, and EMT, especially in obesity-related PC.^424,427^ Moreover, it was shown that inflammatory macrophages can be converted into Ym1^+^ alternatively activated macrophages at ADM/PanIN lesions in the presence of IL-13 and oncogenic *Kras* and then drive pancreatic fibrogenesis and carcinogenesis by releasing IL-1Rα and CCL2.^27^ Furthermore, mutant *KRAS* in organoids was demonstrated to induce a protumorigenic phenotype in macrophages, with these transformed macrophages reducing epithelial PEDF expression and favoring a cancerous phenotype of epithelial cells during the early stage of pancreatic carcinogenesis, thereby promoting neoplastic growth.^428^ Likewise, the extracellular KRAS^G12D^ protein packed into exosomes can be released from cancer cells via autophagy-dependent ferroptosis under oxidative stress and then taken up by macrophages through an AGER-dependent mechanism, leading to the switch of TAMs into an M2-like phenotype via STAT3-dependent FAO.^429^
  Communication between macrophages and adipocytes plays an important role in the pathogenesis of many diseases related to obesity, including DM and PC.^424^ TAMs in patients with obesity and DM can produce substantial proinflammatory cytokines to favor a hypoxic and immunosuppressive TME suitable for pancreatic carcinogenesis, such as IL-1β, IL-6, and TNF-α, activating NF-κB and Hh signaling.^424^ In addition, TAMs are involved in immunoregulation by reshaping the metabolism of the TME. While proinflammatory M2 macrophages fuel the enhancement of glycolysis and FA biosynthesis and the disruption of the TCA cycle, M2 macrophages increase oxidative phosphorylation, FAO, and arginine metabolism.^424^ Numerous preclinical studies have confirmed that TAMs can promote the Warburg effect in the TME of PC via different pathways and contribute to carcinogenesis and cancer progression.^424^
  Apart from being implicated in the production of proinflammatory cytokines and chemokines, the infiltration of macrophages in adipose tissue was also suggested to promote PanIN development and PC initiation.^424^ In detail, the release of proinflammatory cytokines from macrophages increases the levels of FAs in the microenvironment owing to lipolysis, and this process can accelerate pancreatic carcinogenesis. In the conditional *Kras*^*G12D*^ mouse model, mice with diet-induced obesity developed hyperinsulinemia, hyperglycemia, hyperleptinemia, and elevated levels of IGF-1. The pancreas of these animals showed important signs of inflammation with increased numbers of infiltrating inflammatory macrophages and T cells, elevated levels of several cytokines and chemokines, increased stromal fibrosis, and more advanced PanIN lesions.^430^
  Since CAFs in the TME are capable of inducing the polarization of TAMs from the antitumorigenic M1 to the protumorigenic M2 phenotype through the secretion of proinflammatory cytokines and chemokines, polarized M2 TAMs also produce a variety of cytokines to promote EMT, which is beneficial for the spreading of PC cells during the early stage of PC formation.^336,424^ Through the activation of the transcription factor NRF2 and subsequent upregulation of CD163 and Arg1 and downregulation of IL-1β and IL-6, cancer cells induce an M2-like phenotype in TAMs and promote VEGF expression to augment EMT.^431^ Expressing multiple proangiogenic factors (e.g., VEGF-A, TGF-β1, TNF-α, IL-1β, IL-8, IL-10, IL-35, PDGF, and FGF-2) and enhancing the activity of MMPs and thymidine phosphorylase, macrophages function as one of the most important regulators of angiogenesis via the regulation of HIF-1α and participate in the entire angiogenic process, including the initiation, anastomosis, remodeling, maturation and plexus formation of vessels.^424^ As a major source of VEGF, a main driver of angiogenesis, macrophages facilitate angiogenesis in a VEGF-dependent manner in this process. TAMs and VEGFs all have a synergistic effect on enhanced angiogenic activity, which supplies the newly formed PC tumor with adequate blood and oxygen. Moreover, the infiltration of TAMs in the hypoxic TME can also elevate the expression of HIF-1α and VEGF-A.^424^
4. *Mast cells*. Being ubiquitous in the interstitial spaces of the body and residing in the connective tissue close to blood vessels, lymphatic capillaries, nerves, and epithelia, mast cells are known for their roles in allergic and anaphylactic reactions, with their involvement in acute or chronic inflammatory responses endowing them with the ability to participate in carcinogenesis.^432^ It was demonstrated in β-cell tumor models that the activation of MYC in vivo triggers fast recruitment of mast cells to the tumor site, which is necessary for macroscopic tumor expansion, while the inhibition of mast cells induces hypoxia and the death of cancer cells and endothelial cells.^433^ Like macrophages, mast cells can make selective adaptations to secrete pro- or anti-inflammatory molecules according to microenvironmental stimuli. Moreover, mast cells can change the microenvironment by producing cytokines and chemokines that impact fibrogenesis, angiogenesis, and ECM composition,^432^ while they can also be counteractivated by PC cells.^434^
  Mast cells facilitate the invasion of cancer cells, as their infiltration in tumors can promote proliferation and immunoregulation by remodeling the TME.^432^ All parts of the mast cell-mediated inflammatory response participate in the inflammatory response against cancer. In contrast, the epigenetic modifications in response to environmental changes induced by the inflammatory response drive the development of cancer.^432^ In addition to regulating vascular permeability and enhancing tissue healing during inflammation, mast cells also modulate innate and adaptive immunity by tuning the functions and activities of other immune cells.^432^ Mast cells not only take part in innate immunity with neutrophils, macrophages, and NK cells but also regulate the migration of T cells and the growth and differentiation of B cells.^432^ In the case of low-grade chronic inflammatory conditions such as obesity and DM, mast cells synthesize extra proinflammatory signaling molecules to recruit and activate multiple types of leukocytes, including macrophages and lymphocytes, further promoting tissue destruction and inflammation, and the interactions between mast cells and fibroblasts are essential for fibrogenesis in the TME.^432^
  Mast cells can be quite stable in the hypoxic TME, surviving via the release of IL-6, supporting tumor progression and elevating the level of ROS.^432^ Under this condition, cancer cells use mast cells as mediators to regulate the immune response of other immune cells via various cytokines and chemokines [e.g., TNF-α, macrophage inflammatory protein-1 (MIP-1), and MCP]. Nevertheless, similar to many other immune cells, mast cells either positively or negatively regulate the immune response against cancer, enhancing or restraining tumor growth. Accordingly, the actual impact of mast cells may differ depending on the type and phase of the tumor, and it is now generally accepted that different subsets of mast cells infiltrate tumors at different stages.^432^
  More importantly, mast cells contribute to pancreatic carcinogenesis by enhancing angiogenesis^435^ and releasing a broad range of MMPs.^436^ In addition, via interactions with CAFs, mast cells promote fibrosis and angiogenesis by releasing substantial amounts of proangiogenic molecules, including VEGFs, angiopoietin-1, heparin, TNF, and FGF, in PC.^432^ Furthermore, mast cells promote EMT and create the morphogenetic basis favorable for the subsequent evolution of the tumoral interstitium by producing signaling molecules to sustain crosstalk with cancer cells,^432^ through which mast cells facilitate the invasion of CAFs in the tumoral interstitium and the development of a chaotic vasculature (granulation tissue), breeding tumor cells with the resources vital for their growth and metastatic invasion.^432^
  In summary, mast cells support carcinogenesis via a complementary function to maintain the evolution of the tumor, which includes the creation of a tissue interstitium suitable for the implantation and survival of CSCs and the disruption of the immune response in the TME to ensure the proliferation and invasiveness of cancer cells. Finally, a chaotic vascular and fibrotic stroma is established to promote angiogenesis and EMT.^432^ However, at least for now, the roles of mast cells in pancreatic carcinogenesis remain largely unknown.
5. *MDSCs*. As a heterogeneous population of immature myeloid cells (CD15^+^CD11b^+^) accumulating during chronic inflammatory conditions such as cancer, MDSCs can be commonly divided into granulocytic or polymorphonuclear MDSCs (PMN-MDSCs, CD15^+^CD66b^+^) and monocytic MDSCs (M-MDSCs, CD66b^-^), the former are phenotypically and morphologically similar to neutrophils and represent more than 80% of all tumor-associated MDSCs, whereas the latter are phenotypically and morphologically similar to monocytes.^335^ Compared to the absence of MDSC infiltration in normal pancreatic tissues, the number of MDSCs is drastically increased in both the circulation and the TME, which is associated with the cancer stage in human PDAC.^335,415^ Moreover, human and animal studies have shown that GM-CSF and several other chemokines from the CXC family are the principal stimulators of MDSC recruitment and differentiation. At the same time, RAGEs are also likely to be a part of this process and promote pancreatic carcinogenesis and progression.^335,415,437^ Additionally, YAP, which is involved in obesity-related pancreatic carcinogenesis, can also deteriorate immunosuppression by elevating the expression and secretion of cytokines and chemokines that enhance the differentiation and accumulation of MDSCs.^335,438^

The immunosuppressive effects of MDSCs involve their various interactions with other cells within the TME, impairing innate and adaptive immunity against cancer. In detail, MDSCs have been shown to diminish the cytotoxicity of NK cells and promote the polarization of macrophages toward an M2 phenotype. MDSCs also have a suppressive impact on both CD4^+^ and CD8^+^ T cells through direct cell-to-cell contact by upregulating PD-L1 and stimulating the expansion of immunosuppressive Tregs via the IL-10-dependent secretion of TGF-β and IFN-γ,^335^ where a similar effect was also observed in IL-6.^415^ In contrast, the targeted depletion of MDSCs has been shown to reactivate the endogenous antitumor T-cell response and regress *Kras*^*G12D*^-driven PC initiation at early stages.^335^ Recently, a clinical study recruiting patients with premalignant polyps, colon cancer, premalignant IPMN, and PC confirmed a linear increase in the numbers of MDSCs from normal status to premalignancy and cancer. In contrast, no difference in their subpopulation composition or immunosuppressive capacity was noted, suggesting that the external microenvironment rather than the constant phenotypes and properties of MDSCs may play a more significant role in immunosuppression.^439^

**Fig. 8:**
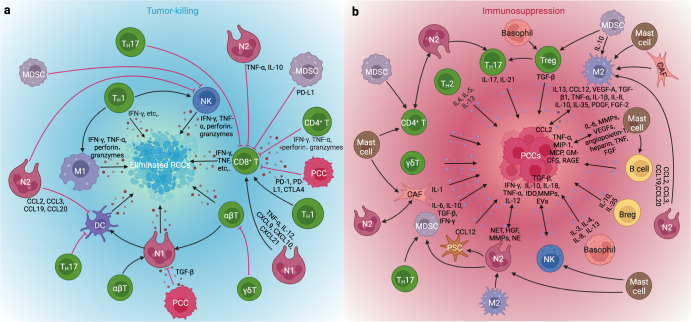
Interactions among cells within the tumor-killing (**a**) and immunosuppressive (**b**) TME of PC. Promotive effects are indicated with black arrows, and the suppressive impacts of cells on anticancer immunity are marked with lines in color. Some signaling components are shown near the arrows and lines. CAF cancer-associated fibroblast, CCL C-C motif chemokine ligand, CTLA4 cytotoxic T lymphocyte-associated protein 4, CXCL CXC-chemokine ligand, DC dendritic cell, EVs extracellular vesicles, FGF fibroblast growth factor, GM-CSF granulocyte-macrophage colony-stimulating factor, HGF hepatocyte growth factor, IDO indoleamine 2,3-dioxygenase, IFN interferon, IL interleukin, M1(2) M1(2) macrophage, MCP monocyte chemoattractant protein, MDSC myeloid-derived suppressor cell, MIP-1 macrophage inflammatory protein 1, MMPs matrix metalloproteinases, N2 N2 neutrophil, NE neutrophil elastase, NET neutrophil extracellular trap, NK natural killer, PC pancreatic cancer, PCCs pancreatic cancer cells, PD(-L) programmed death (ligand), PDGF platelet‐derived growth factor, PSC pancreatic stellate cell, RAGE receptor of advanced glycation end products, TGF transforming growth factor, T_H_ T helper (cell), TNF tumor necrosis factor, Treg regulatory T cell, VEGF vascular endothelial growth factor

Although there are many antitumor immune cells in the TME, their number is far less than that of immunosuppressive cells, which surround the antitumor cells and strongly impair their killing effect, favoring carcinogenesis and cancer progression (Fig. 8).

### Physiological/pathophysiological processes

Obesity and DM are often accompanied by systemic alterations involving almost every aspect of human physiology. In addition to the abovementioned abnormalities, many vital physiological and pathological processes can also participate in pancreatic carcinogenesis. Next, we will summarize several key points that have been intensively studied in recent years.

#### Autophagy

What is mainly regulated by the mechanistic target of rapamycin (mTOR) kinase and the autophagy-related protein (ATG) family is autophagy, a conserved homeostatic process that maintains cellular quality and organ function through the disposal and recycling of cellular components while eliminating cells containing toxic proteins, lipids and organelles.^440^ Given its critical roles in mediating the homeostatic balance, detrimental alterations in autophagy are associated with metabolic disorders and other diseases, including obesity, DM, and cancer.^440,441^ As a unpredictable and dynamic physiological process, autophagy is highly sensitive to changes in nutrients, energy status, the microenvironment (hypoxia, oxidative stress, DNA damage, and protein aggregates), cellular metabolism, inflammatory status, and intracellular pathogens,^442^ and observations of the changes in autophagy in obesity can be highly dependent on the conditions of the models used.^440^ Therefore, it is still controversial whether alterations in autophagy are the cause or the consequence of metabolic disorders in obesity and DM.^440^ Nevertheless, most of the previous findings suggest that autophagy is suppressed under overnutrition conditions such as obesity and DM, although others suggest that autophagy is enhanced in adipose tissue as a compensatory anti-inflammatory response.^440,443^ In addition, the nature of autophagy being influenced by multiple factors results in an interesting scenario in which autophagy can be enhanced and suppressed in the same organ in different models of obesity,^440^ making its roles in metabolic disorders unclear.

Regarding the roles of autophagy in pancreatic carcinogenesis, more than 30 autophagy-related genes and numerous genes with similar functions have been implicated in carcinogenesis,^444^ and previous studies have confirmed that autophagy is vital for the initiation and progression of precancerous lesions in PC. Similar to the proinflammatory effect of suppressed autophagy in obesity,^440^ it was shown that inhibited autophagy could fuel inflammation^445^ and that autophagy participates in AGER-dependent macrophage polarization driven by oxidative stress in PDAC via ferroptosis.^429^ In another study, RAGEs were illustrated to promote cancer cell proliferation during the earliest stages of PC development via the regulation of autophagy, which enhances the IL-6-induced phosphorylation of STAT3.^446^ As one of the regulators of autophagy, ATG5 was also shown to determine the progression and metastasis of PC,^447^ and the knockout of *Atg5* or *Atg7* hindered the progression of PanINs, suggesting that autophagy is necessary for the initiation of malignant transformation.^448^ Likewise, although the decreased tumor growth following the inhibition of autophagy seems to prove its protumorigenic effect, the genetic ablation of autophagy in the pancreas increased tumor initiation but compromised the conversion of these premalignant lesions to invasive cancer and favored prolonged survival.^449^

Additionally, the dual effects of enhanced autophagy on pancreatic carcinogenesis have also been reported. Oncogenic RAS could stimulate autophagy via the BH3-protein Noxa and the autophagy regulator Beclin 1 to improve the survival of Ras-transformed cells by inducing proliferative arrest or premature senescence triggered by autophagy protein ULK3 during oncogene-induced senescence (OIS).^445^ However, it was also suggested that autophagy could prevent carcinogenesis through the clearance of p62, a multidomain signaling adapter protein functioning as a signaling hub in the mediation of cell survival and apoptosis, which can also augment ER stress, oxidative stress, DNA damage, and NF-kB activation.^445^ Similarly, it has been demonstrated that autophagy is protective against carcinogenesis by eliminating excessive ROS.^450^ Although it is easy to reason that suppressed autophagy in obesity and DM may contribute to pancreatic carcinogenesis based on the evidence above, we still cannot draw this conclusion considering the variable characteristics of autophagy and how it is influenced by various factors because it is very likely that different results will be observed using different models under different conditions in vivo and in vitro. Thus, determining the main factors and mechanisms that determine the roles of autophagy in the pathogenesis of diseases should be the focus of future research.

#### ER stress

The ER is involved in the process of protein folding, and ER stress refers to a basic cellular stress response that maintains protein homeostasis under several stressful conditions, such as the accumulation of unfolded proteins in the lumen of the ER. The increased FFA levels in individuals with obesity are associated with ER stress in adipocytes,^451^ which is caused by the increased number of unfolded proteins in the ER owing to the accumulation of ROS, resulting in cell apoptosis eliciting an inflammatory response.^251^ Moreover, WAT inflammation related to ER stress has also been noted in glucose intolerance.^452^ The recognition of ER stress as a factor related to carcinogenesis and cancer development involves the “unfolded protein response (UPR)”. Usually, cells adapt to ER stress by activating the UPR signaling pathway to retain ER homeostasis. Nevertheless, prolonged or severe stress often switches cellular signaling from prosurvival to ER stress-induced apoptosis.^453^ The high demand for protein synthesis in the exocrine pancreas requires the constitutive activation of the UPR to maintain the homeostasis of acinar cells,^454^ which may inevitably induce carcinogenic side effects on these cells of PC origin. In addition, ER stress can induce cellular inflammation and has been demonstrated to be related to pancreatic carcinogenesis. Accompanied by the later increase in UPR proteins, anterior gradient-2 (AGR2), a pro-oncogenic member of the protein disulfide isomerase family of ER-resident proteins, was suggested to be essential for PC initiation following inflammation induced by ER stress.^455^ Driven by ER stress, hypoxia, and ROS, ER oxidoreductase-1α (ERO1L), an ER luminal glycoprotein, is overexpressed in PanINs and PDAC, which promotes tumor growth via the enhanced Warburg effect.^456^ Nevertheless, the cellular response to ER stress is not always carcinogenic,^457^ and the impact of ER stress on tumor growth and the antitumor immune response also makes it a promising target in immunotherapy.^458^

#### Epithelial-mesenchymal transition

The highly fibroinflammatory (or desmoplastic) stroma consisting of a dense ECM is vital for establishing a hypoxic and nutrition-poor TME, promoting the final formation of PC. EMT is a process of epithelial cell differentiation to a mesenchymal phenotype through the loss of epithelial markers such as E-cadherin, certain cytokeratins, occludin, and claudin but the gain of mesenchymal markers such as vimentin, N-cadherin and fibronectin. EMT increases cell motility by lessening cell adhesion to other cells and the matrix to promote the development of PC.^249,336^

Initially, triggered by inflammatory reactions and regulated by numerous signaling pathways, EMT occurs much earlier than the final formation of pancreatic tumors but at the very early stage of carcinogenesis. As a dynamic and reversible process shaped by the TME, EMT endows cancer cells with morphological changes and a higher migratory capability. However, in response to the TME, with its considerable complexity, most cancer cells only undergo “partial” EMT and phenotypic versions instead of a thorough EMT to make adaptations and improve their survival.^336^ However, as mentioned above, EMT is reversible under certain conditions, as cancer cells can revert to an epithelial phenotype. Following the progression of precancerous low-grade neoplastic lesions, various protumoral factors are assembled into the TME, including cytokines and GFs that promote inflammation. At the same time, desmoplastic fibroblasts increase the intertumoral pressure and cause abnormalities in blood vessels, creating a severely hypoxic TME^336^ (detailed in the section *Tumor microenvironment (TME) and cellular perturbations*).

A microenvironment favorable for carcinogenesis exists in animal models of diet-induced obesity characterized by changes in hormones, GFs, cytokines, and adipocytes and alterations in EMT, which can be reversed by calorie restriction (CR), and EMT components may also serve as novel targets for cancer prevention or therapy.^459^ Indeed, enhanced EMT in PC cells is mediated by proinflammatory cytokines secreted in response to obesity,^218^ which is identical for DM, with a desmoplastic stroma and preexisting chronic inflammation promoting neoplastic progression.^336^ As we introduced above, the inflammatory response is a strong promoter of pancreatic carcinogenesis. In turn, precancerous or neoplastic cells can also trigger inflammation in the host immune system, disrupting innate and adaptive inflammatory responses to exacerbate the stromal reaction and further contribute to carcinogenesis via enhanced EMT.^336^

#### Exosome secretion

Transported by a variety of body fluids to distal tissues and organs to activate signaling pathways in target cells, exosomes are a class of nanoextracellular vesicles that function in an autocrine and paracrine manner^460^ and are also produced by stromal and transformed cells in the TME.^461^ Hence, ample evidence has suggested that exosomes play crucial roles in the pathogenesis of multiple physiological disorders and diseases, such as obesity, DM, and PC.^462^ During pancreatic carcinogenesis, exosomes promote the transformation of various precancerous lesions while augmenting angiogenesis, cell migration, and EMT and inhibiting apoptosis.^462^

Sharing similar structural proteins, exosomes secreted by different cell types contain proteins, nucleic acids, lipid molecules, and other inorganic substances. The surface of exosomes contains a variety of lipid raft microdomains that can transduce essential signals, such as signals for apoptosis and cell cycle arrest, via lipid molecules or proteins.^462^ Like exosomal lipids consisting of various components, exosomal nucleic acids consist of almost all kinds of RNAs and several types of DNAs.^462^ Exosomes are released into body fluids upon stimulation by specific signals. Then, they activate the corresponding signaling pathways in target cells, with their production and function varying according to the types and needs of cells.

The validation of targeting oncogenic KRAS using exosomes in PC models provided solid proof that exosomes participate in pancreatic carcinogenesis.^463^ Since exosomes play critical roles in the pathogenesis of obesity and DM,^464^ they can also promote carcinogenesis through various metabolic disorders. A previous study showed that exosomes derived from the adipose tissue of obese rodents can induce the differentiation of peripheral blood monocytes into activated macrophages and stimulate the secretion of proinflammatory IL-6 and TNF-α in a TLR4-dependent manner to fuel insulin resistance.^465^ In addition, obstructive sleep apnea (OSA), a comorbidity commonly seen in patients with obesity as a potential cause of intermittent hypoxia (IH),^462^ was suggested to increase the risk and metastasis of PC.^466,467^ In detail, IH promotes the proliferation and migration of cancer cells and angiogenesis by regulating the exosome content and increasing the production of tumor-promoting exosomes.^462^ In addition, exosomes also promote pancreatic carcinogenesis by mediating the occurrence of both T1DM and T2DM.^462^ In T1DM, apart from enhancing the autoimmune response via the contained proteins functioning as antigens, miRNAs transported by exosomes have also been shown to influence the pathogenesis of T1DM by influencing the apoptosis of β-cells. Similarly, various miRNAs in exosomes can modulate the apoptosis of β-cells in T2DM and control insulin secretion and the formation of pancreatic islets, thereby regulating glucose homeostasis.

In addition to the abovementioned metabolic disorders, exosomes are implicated in the inflammatory reactions of pancreatic carcinogenesis. With an elevated level in the circulation under inflammatory conditions, exosomes lead to pancreatic damage through an action similar to inflammatory factors, mainly characterized by irreversible damage in interstitial fibrosis and parenchymal calcification, where the proinflammatory effects of exosomes originating from different sources vary according to their components.^462^ As a result, pancreatic tissue destruction and exocrine/endocrine deficiency may turn quiescent PSCs with a high proliferative capacity into myofibroblasts, promoting the progression of PanINs.^468^ In particular, exosomal miRNAs can cause extensive fibrosis by promoting desmoplasia,^462^ a common aberrance in the TME that promotes PC development.

Previous studies have shown that exosomes secreted by PC cells can promote the malignant transformation and tumorigenesis of human pancreatic duct epithelial (HPDE) cells via the inhibition of SCL/TAL1 interrupting locus (STIL)^469^ and initiate malignant transformation and carcinogenesis in vivo by inducing random mutations in recipient cells.^470^ Comparisons among human PC cell lines and normal pancreatic epithelial cells confirmed that vesicles from PC cells with enriched proteins contribute to oncogenic cellular transformation. In contrast, vesicles from normal pancreatic cells are abundant in proteins related to the immune response.^471^ To facilitate and maintain PC development, cancer cells produce exosomes to favor an immunosuppressive TME to escape immune surveillance.^472^ DCs are the most critical APCs in the human body, acting through TLRs and producing various ILs, among which TLR4 is particularly indispensable for the antitumor effect of DCs.^462^ However, exosomes produced by PC cells have been shown to suppress the immune response of DCs by increasing intracellular levels of miR-203 and inhibiting the expression of *TLR-4*, *TNF-α*, and *IL-12*.^473^ Moreover, the miR-212-3p^+^ exosomes produced by PC cells were demonstrated to result in the failure of DCs.^474^ PC cells also diminish adaptive and innate antitumor responses by preventing B lymphocytes from recognizing cancer cells and triggering the cytotoxic killing effects of other immune cells.^475^ To create an immunosuppressive TME, exosomes produced by SMAD4-deficient PC cells carrying miR-1260a and miR-494-3p augment cell proliferation and glycolysis.^476^ Exosomes secreted by PC cells in rats can also reduce the proliferation and antiapoptotic capacity of leukocytes and abolish the chemotactic migration of leukocytes to tumor sites,^477^ contributing to tumor formation.

#### Oxidative stress and ROS

Reduction‒oxidation (redox) chemical reactions are a principal constituent of all life. With no exceptions, all normal and neoplastic transformed cells have a redox balance essential for maintaining cell functions. However, incomplete oxygen reduction leads to ROS formation, which is associated with the principle of oxidative stress mediating pathology, as ROS are damaging agents that can structurally and functionally compromise macromolecules, such as nucleic acids, proteins, and lipids. While normal cells have only a limited capacity to maintain the redox balance and nucleotide synthesis, oncogenic transformation can occur once this limit is overcome.^478^ Increased ROS is the direct cause of oxidative stress, and it has been implicated in various conditions such as obesity, DM, and the initiation and progression of PC.^345,479,480^

As a defense against oxidative stress, cells orchestrate a complex network of antioxidants to maintain proper cellular function. Several transcription factors strictly regulate the activity and production of antioxidant equivalents to protect macromolecules from the indiscriminate damage incurred by free radicals.^481^ In particular, nuclear factor erythroid 2-like 2 (NFE2L2/NRF2) is a master regulator of this network and can elevate the expression of genes involved in cytoprotective activities, maintaining redox homeostasis in response to oxidative stress, implicating xenobiotic metabolism, regulating proteasomal subunits and inflammatory response.^481^ In addition, the well-known transcription factor TP53 suppresses ROS accumulation by directly regulating the expression of a variety of antioxidant genes and indirectly inducing the metabolic gene TP53-inducible glycolysis and apoptosis regulator (TIGAR).^481^

ROS represent a double-edged sword in pancreatic carcinogenesis. Even though a low to moderate concentration of ROS in the TME could be adjusted by the antioxidant defense system, they still act as signaling molecules promoting genomic DNA mutation and the proliferation of cancer cells, which initiates neoplastic transformation by activating oncogenes as well as altering gene expression.^345^ Interestingly, an ROS level exceeding the optimum concentration beyond the capacity of the antioxidant defense system leads to irreversible oxidative damage, enhancing cell death via apoptosis, necrosis, and autophagy.^345^ High levels of ROS can cause damage to nucleic acids, thus being carcinogenic as a DNA mutagen or a promoter of genomic instability via the activation of topoisomerase II.^482,483^ On the other hand, cancer cells can enhance ROS generation,^484^ indicating the existence of a vicious cycle in ROS-promoting tumorigenesis. In addition to tumorigenesis, ROS have also been shown to promote cancer cell proliferation, survival, and metastasis in murine models and human cell lines.^485^ Mechanistically, it was suggested that carcinogenesis and its progression driven by ROS are achieved through sustained H_2_O_2_-dependent activation of the PI3K/AKT/mTOR and MAPK/ERK signaling cascades.^481^ Surprisingly, enhanced ROS production also plays a tumor-suppressing role by inducing cell cycle arrest, senescence, and cancer cell death.^481^

Given the biological features of oxidative stress, antioxidant strategies are seemingly a good option for the prevention and treatment of cancer. However, endogenous or exogenous antioxidants all have a dual effect of promoting and suppressing carcinogenesis and progression, similar to oxidative stress. For example, dietary antioxidants are considered cancer-promotive rather than prophylactic in experimental animal models.^481^ Moreover, as two maintainers of redox homeostasis, the elevated expression of *nrf2* and increased intracellular levels of glutathione (GSH) in mice contribute to the initiation and progression of PC.^486^ Similarly, while the high generation of ROS caused by the oncogene *KRAS* increased the proliferation of PC cells and was reversed by manganese superoxide dismutase (MnSOD),^487^ the oxidizing agents, ironically, have been shown to inactivate mitogenic signaling cascades driven by AKT in pancreatic ductal cells.^486^

These self-contradictory results make the experimental evidence regarding the contribution of ROS to carcinogenesis difficult to interpret. Many questions are pending regarding the role of ROS in carcinogenesis, mainly because of the elusive effect of ROS that depends on their origin and cellular location as well as the stage of cancer progression.^481^ This is the main obstacle in implementing antioxidants in cancer prevention and treatment, owing to the topology and temporality of ROS regulation.^488^ In line with this concept, clinical trials have confirmed that antioxidants do more harm than good in cancer prevention.^489^ In this context, with both increased ROS and antioxidants promoting carcinogenesis, we have a long way ahead of us to reveal the mechanisms regulating the distinct response of different cells to oxidative stress, and only when we start to explore the spatial, temporal, and chemical specificity of individual redox couples will we be headed in the right direction to prevent pancreatic carcinogenesis by tackling oxidative stress.

## Closing remarks and perspective

As the two most common and closely related metabolic diseases, obesity and DM are risk factors for many cancers. As the prevalence of obesity and DM is growing rapidly in most parts of the world, increasing attention is being paid to these two diseases and their effects on other diseases. Metabolism is a realm of great significance in cancer research; therefore, the impact of metabolic disorders on carcinogenesis and the progression of cancer has been the focus of scientific research for a long time. Benefitting from these efforts, the great number of publications and emerging novel findings in recent years have deepened our understanding of the relationship between metabolic disorders and carcinogenesis. In this article, we introduced the most studied mechanisms of obesity- and DM-related pancreatic carcinogenesis from several aspects. Therefore, beyond a broader and deeper understanding of the pathogenesis of PC in metabolic disorders, how should we reflect on these findings and what should they bring to patients? We would like to talk about the three most important values of this research field to answer these questions.

The first is the idea of PC prevention. There is no doubt that, if possible, prevention is always the best treatment for every disease. From an epidemiological perspective, the constant rise in the prevalence of obesity and DM is likely to favor a further increase in the occurrence of PC. Currently, the therapeutic options for obesity and DM vary, and their efficacy is promising. Numerous studies have also shown that most treatments for obesity and DM can significantly reduce the risk of PC.^10^ Hence, it is possible that effective prevention or treatment of obesity and DM can benefit patients at a higher risk, which not only will reduce the number of individuals with obesity and DM but also will lighten the future cancer burden. However, if the measures taken to tackle obesity and DM keep failing as they have been, the potentially growing future cancer burden of PC necessitates the translation of the research findings into clinical practice, whether developing sensitive methods to identify PC at an early stage in patients with obesity and DM or at least improving the efficacy of current obesity and DM treatments to prevent long-term lethal outcomes at best. At least for now, detecting early-stage PC in these patients and starting timely interventions to maximize the efficacy of treatments and prolong overall survival seems more ideal and practical than simply attempting to lighten the future cancer burden by tackling obesity and DM.

**Fig. 9:**
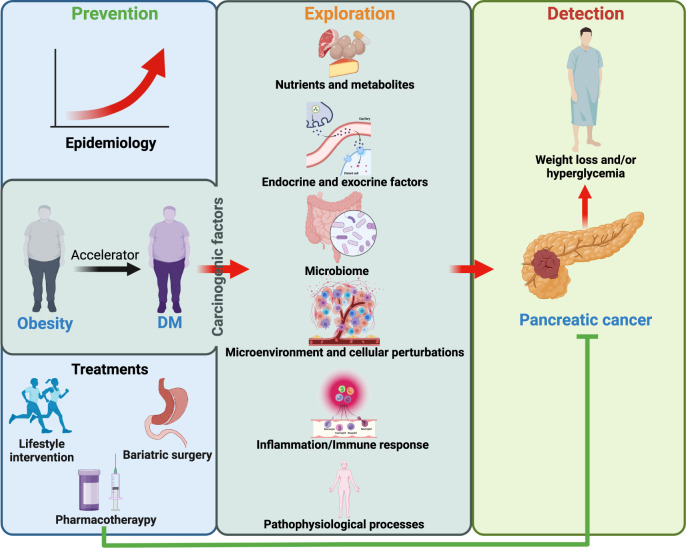
The intricate relationships among obesity, DM, and pancreatic carcinogenesis and the three most important values of relevant research. DM diabetes mellitus

More in-depth exploration of the correlations of obesity, DM, and PC to favor a broader understanding of the mechanisms behind obesity- and DM-related pancreatic carcinogenesis is obviously essential to achieve this goal. Herein, we summarized and introduced the relevant research findings from six aspects (Fig. 9). Considering both the systemic and local influence of obesity and DM on the whole body and the pancreas, pancreatic carcinogenesis could be a multifactorial outcome involving joint input from different culprits. Due to the limited space, it is difficult to be fully comprehensive even if we have included as much varying content as possible, and we must admit that there are some other contributors to obesity- and DM-related pancreatic carcinogenesis that have been missed in this article. Nevertheless, now that we have learned the critical mutations initiating pancreatic carcinogenesis and some of the subsequent genomic alterations,^490^ identifying the enhanced triggering factors that result in modifications of these genes and the maintainers of the premalignant progression in metabolic disorders such as obesity and DM will be a stepstone for future strategies for PC prevention. Therefore, the ultimate challenge in this long journey will be, if it is possible and truly exists, looking for the genuine fuse of this timed bomb or finding the scissor to cut it off.

Finally, and most importantly and realistically, efforts should be made to translate these findings into clinical practice for earlier PC detection. The timing of therapeutic intervention is the decisive factor in the treatment efficacy and prognosis of PC. Even if our abovementioned vision sounds like a daydream, we cannot stop searching for sensitive markers and physiological and biochemical indicators that can be used for surveillance and screening for PC, helping physicians detect early-stage PC in high-risk patients with obesity and DM. In recent years, emerging multiomic studies and advances in sequencing techniques have provided solid evidence on the metabolic landscape of obesity, DM, and other potential triggering factors of pancreatic carcinogenesis from multiple dimensions. The analysis of different clinical samples also hinted at promising directions for experimental practice. In light of these inspirational discoveries, future efforts will breed fruitful results in developing sensitive and cost-effective biomarkers by establishing modified disease models that highly replicate the original and natural pathogenesis through multidisciplinary cooperation and maximum utilization of the available techniques, which we believe serves as the starting point to developing reliable early screening and early diagnostic strategies for PC in populations with obesity and DM in the future.

## Supplementary information

Language Editing Certificate

Change of authorship request form

Publication lisences
