# Supplementary material for: Mechanisms of obesity- and diabetes mellitus-related pancreatic carcinogenesis: a comprehensive and systematic review
Source: Signal Transduct Target Ther. 2023 Mar 24;8:139. doi: 10.1038/s41392-023-01376-w (PMC10039087; doi:10.1038/s41392-023-01376-w)
Supplement: Supplementary file 2 — Change of authorship request form [file 41392_2023_1376_MOESM2_ESM.pdf]

SPRINGER NATURE

Change of authorship request form - Journals (pre-acceptance)

Important information. Please read.

- This form should be used by authors to request any change in authorship (adding/deleting authors) including changes in corresponding authors. This form should not be used for name changes. Please fully complete all sections. Use black ink and block capitals and provide each author's full name with the given name first followed by the family name.
- By signing this declaration, all authors guarantee that the order of the authors are in accordance with their scientific contribution, if applicable as different conventions apply per discipline, and that only authors have been added who made a meaningful contribution to the work.
- Please note, in author collaborations where there is formal agreement for representing the collaboration, it is sufficient for the representative or legal guarantor (usually the corresponding author) to complete and sign the Authorship Change Form on behalf of all authors, **next to the added/removed author(s)**. **(Complete Section 3, followed by Section 6.)**
  In author collaborations where there is no formal agreement for representing the collaboration and **there are more than 10 authors**, one may sign for all, provided the signer appends correspondence that attests that each of the authors have agreed to the change **and the added/removed authors sign the form**. **(Complete Section 3, followed by Section 6.)**
- Please note, we cannot investigate or mediate any authorship disputes. If you are unable to obtain agreement from all authors (including those who you wish to be removed) you must refer the matter to your institution(s) for investigation. Please inform us if you need to do this.
- If you are not able to return a fully completed form within **30 days** of the date that it was sent to the author requesting the change, we may have to withdraw your manuscript. We cannot publish manuscripts where authorship has not been agreed by all authors (including those who have been removed).
- Incomplete forms will be rejected.
- Please return/upload this form, fully completed, to the Journals Editorial Office. The Journal and/or Publisher will consider the information you have provided to decide whether to approve the proposed change in authorship. We may decide to contact your institution for more information or undertake a further investigation, if appropriate, before making a final decision.

SPRINGER NATURE

Change of authorship request form - Journals (pre-acceptance)

Section 1: Please provide the current title of manuscript

Manuscript ID no.: SIGTRANS-08193R1

Title: Mechanisms of obesity- and diabetes mellitus-related pancreatic carcinogenesis: A comprehensive and systematic review

Section 2: Please provide the previous authorship, in the order shown on the manuscript before the changes were introduced. Please indicate the corresponding author by adding (CA) behind the name.

	First name(s)	Family name	ORCID or SCOPUS id, if available
1st author	Rexiati	Ruze	0000-0002-0891-5307
2nd author	Jianlu	Song	0000-0001-7181-2266
3rd author	Xinpeng	Yin	0000-0002-5069-4421
4th author	Yuan	Chen	0000-0003-0449-1356
5th author	Ruiyuan	Xu	0000-0003-4786-6330
6th author	Chengcheng	Wang (CA)	0000-0001-9806-0949
7th author	Qiang	Xu (CA)	0000-0002-6785-7178
8th author			
9th author			
10th author			

Please use an additional sheet if there are more than 10 authors.

SPRINGER NATURE Springer Nature is one of the world’s leading global research, educational and professional publishers, created in May 2015 through the combination of Nature Publishing Group, Palgrave Macmillan, Macmillan Education and Springer Science+Business Media.

SPRINGER NATURE

Change of authorship request form - Journals (pre-acceptance)

Section 3: Please provide a justification for change. Please use this section to explain your reasons for changing the authorship of your manuscript, e.g. what necessitated the change in authorship? Please refer to the (journal) policy pages for more information about authorship. Please explain why omitted authors were not originally included and/or why authors were removed on the submitted manuscript.

Dear editors,

We wish to make a minor change on the corresponding author list in our revised manuscript: Prof. Qiang Xu will be replaced by Prof. Yupei Zhao.

In fact, Prof. Zhao, Prof. Xu, and Dr. Chengcheng Wang co-supervised this whole project, with Prof. Zhao being the senior supervisor. Before our initial submission, Prof. Zhao decided not to be listed, and this is the reason why he was omitted in the first place. Recently we had a team meeting where Prof. Xu suggested that the name of Prof. Zhao should not be omitted given his contribution and he should be entitled as the corresponding author instead of himself. Prof. Zhao and the whole team admired his courtesy and decided to respect this decision after discussion.

We hereby confirm that all authors listed are fully aware of and support the decision to make the above change to the authorship, and we feel deeply sorry for failing to make an earlier request and clarification on this matter.

Section 4: Proposed new authorship. Please provide your new authorship list in the order you would like it to appear on the manuscript. Please indicate the corresponding author by adding (CA) behind the name. <u>If the Corresponding Author has changed, please indicate the reason under section 3</u>.

	First name(s)	Family name (this name will appear in full on the final publication and will be searchable in various abstract and indexing databases)	Affiliated institute	E-mail address
1st author	Rexiati	Ruze	Peking Union Medical College Hospital	rishatruzi@hotmail.com
2nd author	Jianlu	Song	Peking Union Medical College Hospital	SongJianlu2017@hotmail.com
3rd author	Xinpeng	Yin	Peking Union Medical College Hospital	yinxinpeng0222@163.com
4th author	Yuan	Chen	Peking Union Medical College Hospital	chenyuan19961023@hotmail.com
5th author	Ruiyuan	Xu	Peking Union Medical College Hospital	xry970124@163.com
6th author	Chengcheng	Wang (CA)	Peking Union Medical College Hospital	wangchengcheng@pumch.cn
7th author	Yupei	Zhao(CA)	Peking Union Medical College Hospital	zhao8028@263.net
8th author				
9th author				
10th author				

Please use an additional sheet if there are more than 10 authors.

SPRINGER NATURE Springer Nature is one of the world's leading global research, educational and professional publishers, created in May 2015 through the combination of Nature Publishing Group, Palgrave Macmillan, Macmillan Education and Springer Science+Business Media.

SPRINGER NATURE

Change of authorship request form - Journals (pre-acceptance)

Section 5: Author contribution, Acknowledgement and Disclosures. Please use this section to provide a new disclosure statement and, if appropriate, acknowledge any contributors who have been removed as authors and ensure you state what contribution any new authors made (if applicable per the journal or book (series) policy). Please ensure these are updated in your manuscript - after approval of the change(s) - as our production department will not transfer the information in this form to your manuscript.

New acknowledgements:

We regretfully failed to cite many critical and excellent original studies related to the topic of this article due to format constraints. Beyond our cordial apology, we sincerely hope the reviews we cited can make it easier for these studies to be discovered by interested readers. All figures were created on BioRender.com with permission for publication.

New Disclosures (financial and non-financial interests, funding):

The authors declare no competing interests.

New Author Contributions statement (if applicable per the journal policy):

R.R., and C.W. conceptualized and designed the article. R.R., J.S., and X.Y. prepared the initial manuscript. Y.C. and R.X. performed literature categorization and helped with the illustrations. C.W. and Y.Z. critically reviewed and supervised the revision of the manuscript. All authors have read and approved the final version of the submitted and published article.

State 'Not applicable' if there are no new authors.

SPRINGER NATURE Springer Nature is one of the world's leading global research, educational and professional publishers, created in May 2015 through the combination of Nature Publishing Group, Palgrave Macmillan, Macmillan Education and Springer Science+Business Media.

SPRINGER NATURE

Change of authorship request form - Journals (pre-acceptance)

Section 6: Declaration of agreement. *All* authors, unchanged, new and removed *must* sign this declaration.
(NB: Please print the form, (docu)-sign and return/upload a scanned copy. Please note that signatures that have been inserted as an image file are acceptable as long as it is handwritten. Typed names in the signature box are unacceptable.) *** Please delete as appropriate. Delete all of the bold if you were on the original authorship list and are remaining as an author.**

	First name	Family name		Signature	Date
1st author	Rexiati	Ruze	I agree to the proposed new authorship shown in section 4 **/and the addition/removal*of my name to the authorship list** /and the proposed change in corresponding author	Rexiati Ruze	2023.01.28
2nd author	Jianlu	Song	I agree to the proposed new authorship shown in section 4 **/and the addition/removal*of my name to the authorship list** /and the proposed change in corresponding author	Jianlu Song	2023.01.29
3rd author	Xinpeng	Yin	I agree to the proposed new authorship shown in section 4 **/and the addition/removal*of my name to the authorship list** /and the proposed change in corresponding author	Xinpeng Yin	2023.01.28
4th authors	Yuan	Chen	I agree to the proposed new authorship shown in section 4 **/and the addition/removal*of my name to the authorship list** /and the proposed change in corresponding author	Yuan Chen	2023.01.30
5th author	Ruiyuan	Xu	I agree to the proposed new authorship shown in section 4 **/and the addition/removal*of my name to the authorship list** /and the proposed change in corresponding author	Ruiyuan Xu	2023.01.30
6th author	Chengcheng	Wang	I agree to the proposed new authorship shown in section 4 **/and the addition/removal*of my name to the authorship list** /and the proposed change in corresponding author	Chengcheng Wang	2023.01.30
7th author	Yupei	Zhao	I agree to the proposed new authorship shown in section 4 **/and the addition/removal*of my name to the authorship list** /and the proposed change in corresponding author	Yupei Zhao	2023.01.31

SPRINGER NATURE Springer Nature is one of the world's leading global research, educational and professional publishers, created in May 2015 through the combination of Nature Publishing Group, Palgrave Macmillan, Macmillan Education and Springer Science+Business Media.

Change of authorship request form - Journals (pre-acceptance)

	First name	Family name		Signature	Date
8th author			I agree to the proposed new authorship shown in section 4 /**and the addition/removal*of my name to the authorship list** /and the proposed change in corresponding author		
9th author			I agree to the proposed new authorship shown in section 4 /**and the addition/removal*of my name to the authorship list** /and the proposed change in corresponding author		
10th author			I agree to the proposed new authorship shown in section 4 /**and the addition/removal*of my name to the authorship list** /and the proposed change in corresponding author		

Please use an additional sheet if there are more than 10 authors.

In case of author collaborations with formal agreement:

	Name of consortium/consortia	First name	Family name		Signature	Date
Representative/legal guarantor				I agree to the proposed new authorship shown in section 4 /**and the addition/removal*of my name to the authorship list** /and the proposed change in corresponding author		

Both added/removed authors should complete the information in the first table under Section 6.

---- End of form ----

SPRINGER NATURE Springer Nature is one of the world’s leading global research, educational and professional publishers, created in May 2015 through the combination of Nature Publishing Group, Palgrave Macmillan, Macmillan Education and Springer Science+Business Media.
